# The role of GABA_B_ receptors in the subcortical pathways of the mammalian auditory system

**DOI:** 10.3389/fendo.2023.1195038

**Published:** 2023-08-11

**Authors:** Rostislav Tureček, Adolf Melichar, Michaela Králíková, Bohdana Hrušková

**Affiliations:** ^1^ Department of Auditory Neuroscience, Institute of Experimental Medicine, Academy of Sciences of the Czech Republic, Prague, Czechia; ^2^ Second Faculty of Medicine, Charles University, Prague, Czechia

**Keywords:** GABAB receptor, auditory, synaptic transmission, neuronal excitability, hearing loss, tinnitus

## Abstract

GABA_B_ receptors are G-protein coupled receptors for the inhibitory neurotransmitter GABA. Functional GABA_B_ receptors are formed as heteromers of GABA_B1_ and GABA_B2_ subunits, which further associate with various regulatory and signaling proteins to provide receptor complexes with distinct pharmacological and physiological properties. GABA_B_ receptors are widely distributed in nervous tissue, where they are involved in a number of processes and in turn are subject to a number of regulatory mechanisms. In this review, we summarize current knowledge of the cellular distribution and function of the receptors in the inner ear and auditory pathway of the mammalian brainstem and midbrain. The findings suggest that in these regions, GABA_B_ receptors are involved in processes essential for proper auditory function, such as cochlear amplifier modulation, regulation of spontaneous activity, binaural and temporal information processing, and predictive coding. Since impaired GABAergic inhibition has been found to be associated with various forms of hearing loss, GABA_B_ dysfunction could also play a role in some pathologies of the auditory system.

## 1 Introduction

Gamma-aminobutyric acid type B receptors (GABA_B_Rs) are G-protein coupled receptors (GPCR) for GABA, which together with glycine represent the major inhibitory transmitters in the mammalian nervous system. They are widely distributed in nervous tissue, where they regulate neuronal excitability, oscillatory activity and neurogenesis, and are involved in processes such as synaptic plasticity, memory formation and nociception (reviewed in (1–7). Alterations in GABA_B_ functions have been linked to a variety of neurological states and psychiatric disorders including drug addiction, anxiety, cerebral ischemia, depression, epilepsy, neuropathic pain, and spasticity, and Alzheimer’s disease (7–10). In the auditory system, GABA_B_R subunit expression generally shows high levels, particularly in the cochlea, cochlear nucleus, inferior colliculus, medial geniculate nucleus and auditory cortex (11–18). Increasing evidence suggests their functional involvement in the neural circuits that make up these areas, and it is emerging that dysfunction of GABA_B_Rs could play a role in some pathologies of the auditory system. In this review, we summarize the current knowledge on the distribution and functions of GABA_B_Rs in the peripheral and subcortical parts of the central auditory system obtained in animal studies, mostly using various rodent models.

## 2 Structural basis for heterogeneity in GABA_B_R functions

**Figure 1:**
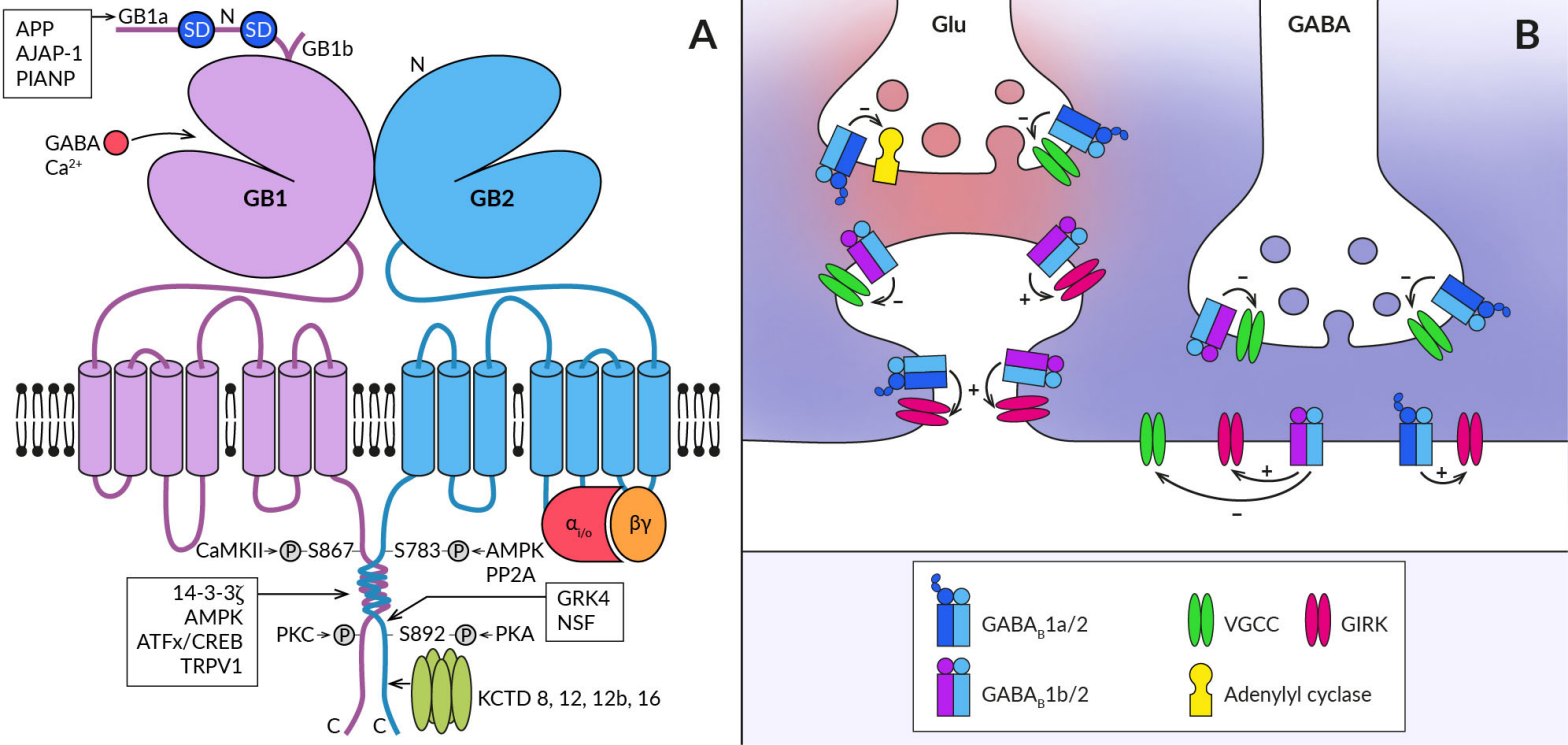
Schematic representation of the GABA_B_R heterodimer and coupling of its subtypes to effectors at central synapses. **(A)** The functional GABA_B_R consists of two subunits, GABA_B1_ (GB1) and GABA_B2_ (GB2). Both subunits contain large extracellular N-terminal domains (N), seven transmembrane domains connected by three intracellular and three extracellular loops, and an intracellular C-terminus. The N-terminal domain of GABA_B1_ contains a binding site for the agonist (GABA) and for the endogenous positive allosteric modulator (Ca^2+^). The two most common splice variants of GABA_B1_ (GB1a and GB1b) differ in the presence of two sushi domains (SD) at the N-terminus of GABA_B1a_. Sushi domain binding proteins, the β-amyloid precursor protein (APP), the adherence junction-associated protein 1 (AJAP-1), and the PILRα-associated neural protein (PIANP) form complexes with GABA_B1a/2_ receptors. The GABA_B2_ subunit interacts with heterotrimeric G-proteins (αi/o, βγ) and stimulates their activation. At least four phosphorylation sites were identified on GABA_B_R subunits: S867 and a yet unidentified site at the C-terminus on the GABA_B1_ subunit are phosphorylated by calcium/calmodulin-dependent protein kinase II (CaMKII) and protein kinase C (PKC), respectively; on the GABA_B2_ subunit, S783 is phosphorylated by 5′AMP-dependent protein kinase (AMPK) and S892 is phosphorylated by protein kinase A and dephosphorylated by protein phosphatase 2A (PP2A). Other GABA_B1_ interacting proteins include 14-3-3 proteins, the capsaicin receptor TRPV1 and ATF/CREB family transcription factors. By specifically interacting with the latter, GABA_B_R can directly influence gene expression. GABA_B2_ can further associate with G-protein receptor kinase 4 (GRK4) and N-ethylmaleimide-sensitive factor (NSF), leading to the regulation of GABA_B_R activity. The C-terminus of GABA_B2_ contains a binding site for auxiliary receptor subunits, proteins of the potassium channel tetramerization domain (KCTD) family. **(B)** GABA_B_Rs are expressed in presynaptic and postsynaptic compartments of both excitatory (Glu) and inhibitory (GABA) synapses (synaptic glutamate and GABA are shown in red and blue, respectively). They associate with effector enzymes and ion channels (adenylyl cyclase, VGCC, GIRK) to regulate neurotransmitter release and neuronal excitability. GABA_B1a_-containing receptors (GABA_B_1a/2) are preferentially localized in the presynaptic membrane of both types of synapses and less frequently at postsynaptic sites such as the dendritic shaft or spine neck. In contrast, GABA_B1b_-containing receptors (GABA_B_1b/2) prefer postsynaptic sites but are also expressed in inhibitory terminals. GABA_B_Rs at inhibitory synapses are activated by synaptic GABA and can mediate slow inhibitory postsynaptic currents. Heteroreceptors at excitatory synapses are activated either tonically by ambient agonist concentration or require GABA spillover from neighboring inhibitory synapses.

GABA_B_Rs are obligatory heteromers of GABA_B1_ and GABA_B2_ subunits (19) (**Figure 1A**). The subunits heterodimerize through a C-terminal coiled-coil domain which displaces an ER retention signal protein from GABA_B1_, thus allowing expression of the assembled complexes on the plasma membrane (20, 21). Despite their structural similarity, the subunits play different functional roles in the activation of the receptor heteromer. During this process, GABA_B1_ mediates agonist binding to GABA_B_R through its N-terminal ‘flytrap’ domain. This induces a series of conformational changes of the receptor and activation of the G_i/o_ protein *via* GABA_B2_ intracellular loops (for a review, see (22–24). Upon activation, the G protein G_α_ subunits inhibit adenylyl cyclase to decrease cytosolic cAMP levels while the G_βγ_ subunits inhibit voltage-gated Ca^2+^ channels (VGCC) or open inwardly rectifying Kir_3_ K^+^ channels (GIRK) (25–27). Through coupling to these effector enzymes and ion channels, GABA_B_Rs act as important regulators of neurotransmitter release, neuronal excitability and propagation of dendritic spikes (28–32) but see (33).

The physiological functions of GABA_B_Rs critically depend on their density and location in specific neural compartments, as well as on the kinetics of their signaling. These properties of GABA_B_R are, on the other hand, significantly modulated by its post-translational modifications and interactions with associated proteins. First, ample evidence exists that phosphorylation of GABA_B_R subunits by serine/threonine protein kinases bidirectionally affects both cell surface receptor expression and the magnitude of its responses (for details, see (34, 35). Phosphorylation of GABA_B1_ at S867 by calcium/calmodulin-dependent kinase II or at an unidentified site by protein kinase C triggers GABA_B_R internalization or desensitization (36, 37). Conversely, phosphorylation of GABA_B2_ by adenosine monophosphate-activated protein kinase (S783) or cAMP-dependent protein kinase (S892) stabilizes GABA_B_Rs at the cell surface (38, 39). Second, the subcellular localization of GABA_B_R and the dynamics of its intracellular trafficking depend, at least in some brain areas, on the type of GABA_B1_ isoform (**Figure 1B**). The GABA_B1a_ variant containing two extracellular sushi domains (SDs) at its N-terminus is predominantly expressed in glutamatergic axonal terminals whilst the GABA_B1b_ lacking SDs expresses in somatodendritic parts (31, 40–42). GABA_B1a_ is also present in dendritic shafts but seems to be excluded from the spine heads (40). Recent studies have identified several proteins that interact with SDs (**Figure 1A**) and promote axonal trafficking of GABA_B1a_ and/or receptor stabilization at the presynaptic plasma membrane (43–45). Among these proteins, the amyloid-precursor protein (APP) appears to play a key role, linking the APP/GABA_B_R complex to the axonal trafficking motor (for details, see (19, 23). Moreover, a secreted cleaved APP fragment has been shown to regulate synaptic transmission in the hippocampus of mice in a GABA_B_R-dependent manner (45), but it appears to act through a more complex mechanism than as a functional receptor ligand (46). GABA_B1_ subunits have also been reported to associate with 14-3-3 proteins and the capsaicin receptor TRPV1 (47, 48), with both interactions being implicated in chronic pain conditions. 14-3-3 binds to the C-terminus of GABA_B1_ to dissociate the GABA_B_ heterodimer, resulting in impaired GABA_B_ signaling and reduced control of TRPV1 sensitization in spinal neurons (47–49).

Lastly, four cytosolic K^+^-channel tetramerization-domain (KCTD) proteins KCTD8, KCTD12, KCTD12b and KCTD16, which constitutively bind to the GABA_B2_ C-terminal domain as pentamers, increase receptor surface expression and show multiple effects on its response kinetics (50–52). The KCTD proteins comprise the N-terminal T1 and C-terminal H1 domains (53, 54), capable of simultaneous interactions with GABA_B2_ and G_βγ_ subunits of the G protein, respectively (55, 56). The preassembled complex is characterized by elevated potency and accelerated kinetics of G-protein signaling (50, 55). In addition, KCTD12 and 12b induce pronounced desensitization of GABA_B_R responses by activity-dependent stripping of G_βγ_ from GIRK or VGCC channels (55, 56). The desensitization is in turn regulated by phosphorylation of serine-892 on GABA_B2_ or by heteromerization of KCTD12 with KCTD16 (57). KCTD16 itself slows down the deactivation phase of GABA_B_R activated GIRK currents by unknown mechanism (58). Moreover, KCTD8 and KCTD16 contain a C-terminal H2 domain that binds secondary GABA_B_R interacting proteins, such as VGCC or hyperpolarization-activated cyclic nucleotide-gated channels (43). Because many neurons in the brain simultaneously express several KCTDs and some of them possibly all KCTDs, assembly of distinct KCTDs at the receptor increases the molecular and functional repertoire of native GABA_B_Rs (58, 59).

## 3 Cochlear GABA_B_Rs

**Figure 2:**
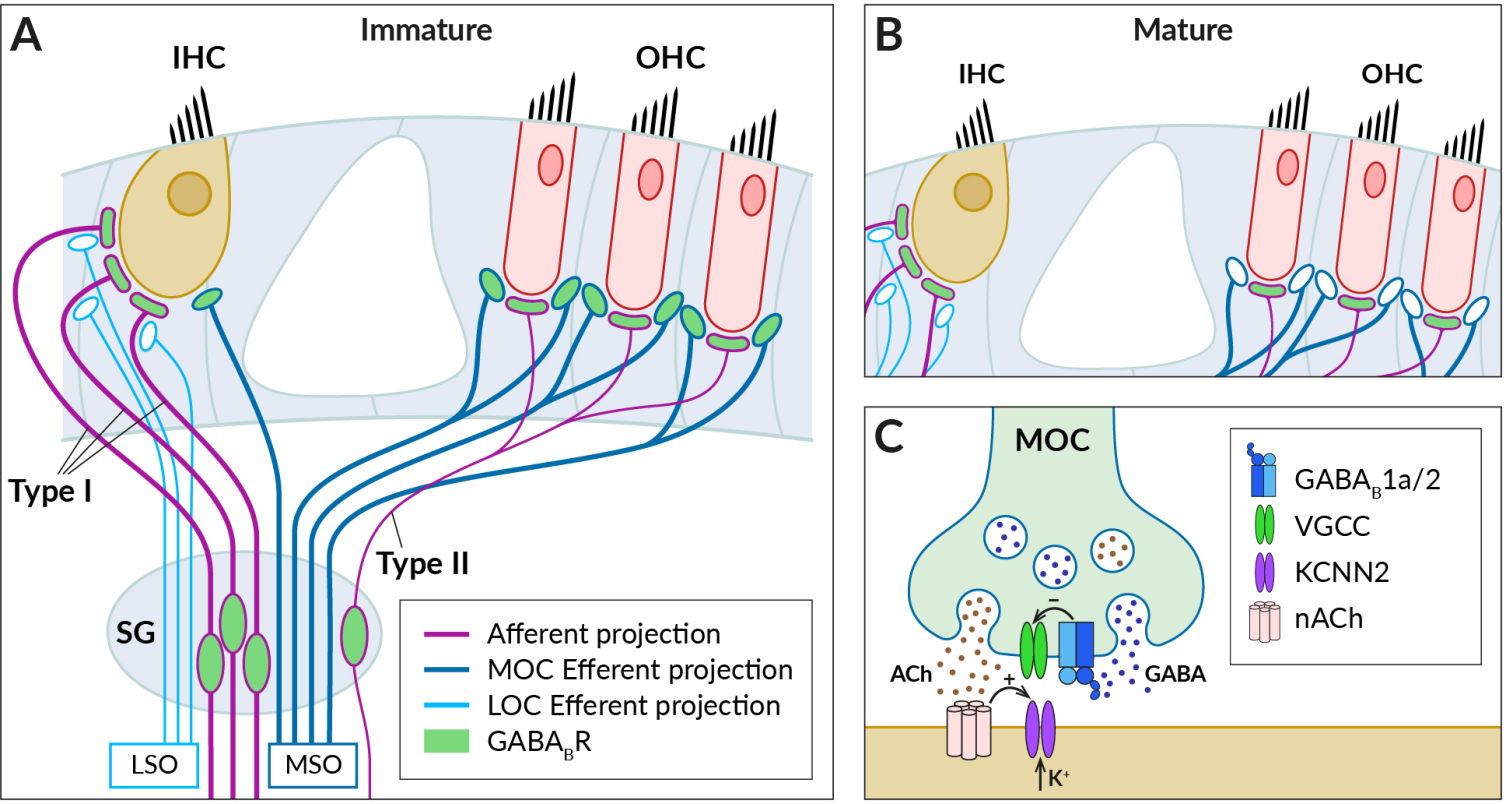
GABA_B_Rs expressed in fibers innervating cochlear hair cells. **(A)** Schematic of afferent (purple) and efferent (blue) innervation of inner (IHC) and outer hair cells (OHC) in the immature cochlea showing the known localization of the GABA_B_R (green). Receptors have been found in type I and II spiral ganglion (SG) neurons and their afferent terminals on the IHC and OHC, where they are thought to regulate glutamate-evoked responses, and in the terminals of the medial olivocochlear bundle (MOC), which originates around the medial superior olive (MSO). The presence of GABA_B_Rs in fibers of the lateral olivocochlear bundle (LOC) arising from the lateral superior olive (LSO) was not detected. **(B)** In the adult cochlea, GABA_B_Rs disappear from the terminals of efferent fibers, along with efferent innervation of IHC by MOC fibers. **(C)** Schematic representation of the cholinergic synapse formed by MOC fibers on the IHC somata. At this synapse, GABA_B_Rs control the secretion of acetylcholine (ACh, brown dots), which is released together with GABA (blue dots) from the MOC terminal. GABA is thought to activate presynaptic GABA_B1a/2_ autoreceptors, which regulate acetylcholine release by inhibiting presynaptic VGCC channels. Acetylcholine binds to nicotinic receptors (nACh) on the IHC, which elicit postsynaptic Ca^2+^ transients and K^+^ currents *via* Ca^2+^-dependent K^+^ channels (KCNN2) (see section 3 for further details).

In the mammalian cochlea, the sensory organ of Corti comprises one row of inner and three rows of outer hair cells (IHCs and OHCs) (**Figure 2**). Their main tasks are to amplify the incoming auditory signals (OHC) and to relay auditory information to the brain (IHC) (for a review, see (60, 61). Hair cells receive both afferent and efferent innervation. The afferent innervation is carried by spiral ganglion neurons type I and II, which respond to glutamate released by IHCs and OHCs, respectively, and initiate and conduct action potentials to the cochlear nucleus (62–65). GABA_B_Rs have been found in both type I and type II spiral ganglion neurons and their afferent terminals at IHCs and OHCs that do not express GABA_B_Rs themselves (66–70). Activation of the receptors with baclofen diminished glutamate induced increases of intracellular Ca^2+^ concentration in cultured spiral ganglion neurons (66). The mechanism underlying the inhibition has not been revealed but these observations would be consistent with GABA_B_R-mediated regulation of the excitability of IHC afferents by reducing the Ca^2+^ permeability of postsynaptic NMDA receptors (32, 71). In addition, GABA_B_Rs could also inhibit Ca^2+^-dependent K^+^ conductance in a way found in vestibular hair cell afferents (72–75), thereby modulating the spike frequency in spiral ganglion neurons (73, 76).

The efferent fibers originate from the superior olivary complex and allow feedback control of cochlear activity by the auditory brainstem (for reviews, see (77–79). There are two groups of olivocochlear efferents (OC): medial OC (MOC), large-diameter myelinated fibers arising from regions near the medial superior olive (MSO) and releasing acetylcholine to reduce the gain of the OHC amplifier, and lateral OC (LOC), thinner unmyelinated fibers that arise near the lateral superior olive (LSO) and modulate the excitability of type I afferents by releasing multiple transmitters, including acetylcholine and GABA. Functional GABA_B_Rs have been found in OC bundles, where their expression appeared to be developmentally regulated (80) (**Figure 2**). Mice during the second postnatal week express GABA_B1a/2_ receptors at axonal terminals of OC fibers innervating somata of OHCs and, at this developmental stage, also IHCs (68) (**Figure 2A**). The receptors control acetylcholine release at efferent synapses *via* inhibition of presynaptic P-/Q-type VGCC (68) (**Figure 2C**). In adult mice, however, no GABA_B_Rs or their disinhibitory effects were observed in MOC terminals or OHCs, respectively (67) (**Figure 2B**).

The role of cochlear GABA_B_Rs in the auditory function remains to be understood. The receptors are thought to be activated *in vivo* by GABA co-released with acetylcholine from LOC and MOC fibers (79–81) (**Figure 2C**). The firing frequency of the fibers and thus the secretory activity of their efferent synapses depend on the intensity of sound stimulation (77, 82). During low-intensity acoustic stimulation, GABA_B_Rs can be activated predominantly by the background GABA (83) and tonically suppress the release of acetylcholine at OC terminals. Consistent with this expectation, selective GABA_B_R antagonists increase the amplitudes of postsynaptic currents elicited in hair cells by low-frequency stimulation of MOC fibers (68). The low basal probability of acetylcholine release then allows MOC-OHC synapses to respond to high-frequency stimulation by strongly facilitating cholinergic postsynaptic currents (78, 84). It has been proposed that the presynaptic facilitation together with a summation of repetitive postsynaptic currents significantly increase the reliability and strength of cholinergic synaptic transmission during high-intensity acoustic stimulation (78). This, in turn, would lead to a greater reduction in the gain of the OHC amplifier when exposed to intense sounds and to the protection of the immature cochlea from acoustic trauma (80). Adult mice with deleted GABA_B_Rs exhibit increased hearing thresholds as measured by auditory brainstem responses and distortion product otoacoustic emissions (67). This indicated the importance of GABA_B_Rs for the normal function of the mature cochlea. It has been suggested that deletion of GABA_B_Rs in spiral ganglion and brainstem neurons led to increased spontaneous activity and elevation of the thresholds (67).

Finally, GABA_B_R-associated KCTD12 proteins have been found to be highly expressed in the mammalian cochlea during the early developmental stages (11, 59). Immunostaining experiments located KCTD12 in spiral ganglion neurons and also in cochlear supporting cells and fibrocytes (11). As these non-neuronal cells have been implicated in the K^+^ recycling pathways, KCTD12 could play a role in ion transport or ionic content regulation in the cochlea (11, 85, 86).

## 4 GABA_B_Rs in the auditory brainstem and midbrain

### 4.1 The cochlear nucleus

**Figure 3:**
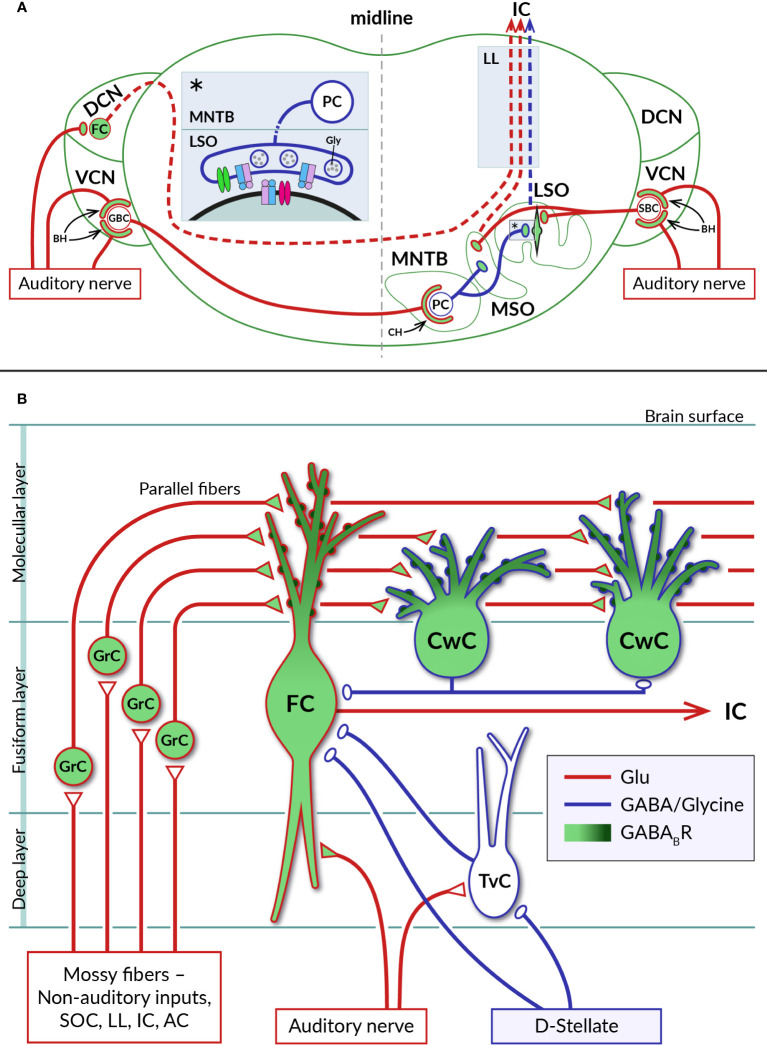
Distribution of GABA_B_Rs in auditory brainstem nuclei. **(A)** Diagram of a coronal section through the brainstem showing a simplified representation of the circuits formed by neurons of the auditory nuclei, dorsal and ventral cochlear nuclei (DCN, VCN), lateral and medial superior olive (LSO, MSO), and medial nucleus of the trapezoid body (MNTB). Excitatory (glutamatergic) and inhibitory (glycinergic) projections are represented by red and blue lines, respectively. GABA_B_Rs have been identified on large axosomatic terminals on spherical bushy cells (SBC) and globular bushy cells (GBC) in the VCN and on MNTB principal cells (PC), referred to as endbulbs of Held (BH) and calyces of Held (CH), respectively, as well as on principal neurons in the LSO and MSO and their excitatory and inhibitory boutons. Dashed lines indicate ascending projections of brainstem neurons passing through the lateral lemniscus (LL) to the inferior colliculus (IC). The inset (asterisk) on the left of the section shows a detail of the inhibitory synapse formed by the axon of an MNTB PC on the soma of an LSO neuron. At mature LSO synapses, presynaptic GABA_B_Rs control glycine release by inhibiting VGCC (green), whereas at immature synapses, somatic GABA_B_Rs additionally regulate postsynaptic excitability by activating GIRK channels (red). See sections 4.1 and 4.2 for details. **(B)** Schematic representation of selected synaptic connections between excitatory (red) and inhibitory (blue) neurons in the DCN (adapted from (87). GABA_B_Rs have been found in pre- and postsynaptic compartments of DCN neurons, where they control glutamate release and short-term synaptic plasticity or neuronal excitability. Presynaptic receptors are localized in auditory nerve endings on basal dendrites of glutamatergic fusiform cells (FC) and in glutamatergic terminals of axons of granule cells (GrC), parallel fibers, innervating apical dendrites of both fusiform cells and glycinergic cartwheel cells (CwC). The subcellular distribution of postsynaptic GABA_B_Rs expressed by fusiform and cartwheel cells shows a dendrosomatic gradient, with receptor density reaching highest values in the distal parts of apical dendrites (dark green). AC – auditory cortex, IC – inferior colliculus, LL – lateral lemniscus, SOC – superior olivary complex, TvC – tuberculoventral cell, D-stellate – a subtype of inhibitory neuron in VCN.

The cochlear nucleus (CN) is the first processing station of the central auditory pathway. It consists of two distinct regions, the dorsal (DCN) and ventral (VCN) cochlear nuclei (**Figure 3**). Fibers of the auditory nerve in both parts contact the major projection neurons and several types of interneurons to distribute sensory information while maintaining the tonotopic organization. Incoming cochlear signals are then preprocessed by CN circuitry, integrated with signals received by multimodal inputs and conveyed to multiple ascending auditory pathways (88–90). In this way, the CN plays a role in processes such as frequency representation, intensity and time coding, localization of sound sources and filtering of self-generated sounds (91).

The existence of functional GABA_B_Rs in the CN was suggested in the pioneering work of Caspary and colleagues (92). Subsequently, GABA_B_Rs were found to be involved in CN circuits at pre- and postsynaptic sites, where they control neurotransmitter release, neuronal excitability and short-term plasticity of synaptic currents. Consistent with the expression of GABA_B_Rs by spiral ganglion neurons, the receptors have been found at the axonal terminals of the auditory nerve on CN neurons (16, 93) (**Figure 3A**). In the VCN, type I fibers contact glutamatergic spherical and globular bushy cells, T-stellate neurons, octopus cells, and glycinergic D- and L- stellate interneurons (94–98). GABA_B_ function was mostly studied in the endbulb of Held synapses formed by auditory nerve fibers on somata of bushy cells. Receptor activation has been found to inhibit presynaptic Ca^2+^ influx through N- or P-/Q-type VGCCs, leading to reduced glutamate release from endbulbs and diminished amplitudes of excitatory postsynaptic potentials in bushy cells (99–101). As a result, GABA_B_R activation reduced the probability of bushy cells to initiate action potentials in response to auditory nerve stimulation. Cell firing could be restored when two converging synaptic inputs were activated simultaneously or by postsynaptic depolarization by group I metabotropic glutamate receptors (99, 100). Thus, in the presence of GABA, bushy cells appeared to function as coincidence detectors with the spiking probability dependent on the synchronous activity of multiple inputs or on modulation *via* other G-protein-dependent pathways. It has been proposed that the action of presynaptic GABA_B_Rs at the endbulb of Held synapses suppresses the relaying of incoming spontaneous activities and enhances the temporal coding observed in bushy cells *in vivo* (99, 102, 103). Potential sources of endogenous agonists for these receptors could include intrinsic and extrinsic inhibitory synaptic inputs to bushy cells arising from D- or L- stellate cells in the VCN, GABA/glycinergic neurons in the DCN and descending inhibitory projections from the superior olivary complex (97, 104, 105). Accordingly, a pharmacological study has shown that repetitive stimulation of inhibitory synapses formed by D-stellate interneurons on bushy cell bodies leads to GABA accumulation, activation of presynaptic GABA_B_ autoreceptors, and suppression of glycinergic postsynaptic currents (106). However, experimental evidence that GABA, which is released from these synapses along with glycine, also actually activates GABA_B_ heteroreceptors on endbulbs has not yet been obtained.

In the DCN, type I fibers innervate basal dendrites of glutamatergic fusiform cells, giant cells and glycinergic tuberculoventral neurons (107) (**Figure 3B**). Type II fibers terminate on neurons in the granular cell domain (65, 108) that receive additional inputs from somatosensory and motor systems (109, 110). The axons of granule cells then enter the molecular layer of the DCN and give rise to parallel fibers that excite the spiny apical dendrites of fusiform cells, as well as GABA/glycinergic cartwheel cells, stellate cells and Golgi cells (87). Fusiform cells, the principal DCN projection neurons, thus represent highly integrative units of the ascending auditory system (89). Both types of excitatory synaptic inputs to these cells were found to be controlled by presynaptic GABA_B_Rs. Activation of the receptors by baclofen reduced the release of glutamate while decreasing short-term synaptic depression at auditory nerve endings and enhancing the facilitation of release from parallel fibers (93, 111). It has been suggested that in this way presynaptic GABA_B_Rs support the sustained transmission of auditory signals to fusiform cells at increased sound intensities and amplify somatosensory information at the parallel fiber synapses formed by high frequency inputs (93). In addition, the excitability of fusiform cells has been found to be regulated by postsynaptic GABA_B_Rs coupled to GIRK and N-type VGCC (93, 112). The concentration of these receptors in the cell membrane shows a dendrosomatic gradient, reaching the highest levels in the distal parts of the apical dendrites (15). This indicates that postsynaptic GABA_B_Rs could control synaptic inputs from parallel fibers more strongly than those from the auditory nerve.

GABA_B_Rs regulating fusiform cell activity are thought to be stimulated by agonists released from inhibitory projections that derive from the superior olivary complex, lateral lemniscus or from local GABA/glycinergic interneurons (**Figure 3**) (113–115). Of the latter, cartwheel cells are the most abundant inhibitory interneurons in the DCN (116). Their excitation through parallel fiber synapses has been shown to cause a strong inhibition of fusiform cells as well as other cartwheel cells (116–118). They are therefore considered to form the basis for both the feed-forward inhibitory and disinhibitory circuits in the DCN (116, 119, 120). Neuronal processes operating in these circuits likely involve GABA_B_R activity. Cartwheel cells have been found to be highly GABA_B1_-immunoreactive, similar to fusiform cells (15, 121, 122), although the functional properties of their GABA_B_Rs remain unexplored. The receptors could regulate spontaneous activity of cartwheel cells in a way observed for other GPCRs which use the same signaling pathway. Kuo and Trussell (123) found that α2 adrenergic receptors, by eliminating the background spiking of cartwheel cells, relieved their inhibitory synapses from depression, thereby enhancing the stimulus-evoked inhibition of fusiform cells.

### 4.2 Superior olivary complex

The superior olive complex (SOC) consists of a group of interconnected brainstem nuclei that process binaural information necessary for sound source localization and modulate the function of other auditory areas *via* the olivocochlear bundle or numerous inhibitory projections (77, 124, 125). Sound-localizing SOC circuits include principal neurons in the medial nucleus of the trapezoid body (MNTB) that convert excitatory signals from the contralateral VCN into properly timed glycinergic inhibition transmitted to the ipsilateral LSO and MSO (126) (**Figure 3A**). GABA_B_Rs have been shown to participate in sound localization mechanisms by modifying the sensitivity of LSO and MSO neurons that encode interaural sound level and time differences (ILDs and ITDs) (see reviews (127, 128), for details). In summary, it has been shown in the gerbil that repeated activation of principal neurons in the LSO and MSO leads to the release of GABA, which then differentially inhibits glutamatergic and glycinergic inputs to these neurons *via* presynaptic GABA_B_Rs (129–131). In both nuclei, GABA is released in an activity-dependent manner, either directly from somatodendritic parts of LSO principal cells or from GABAergic projections to the MSO, allowing feedback control of spiking of LSO and MSO neurons (129, 130). As a result, ILD and ITD responses of neurons show a dependence on their previous spiking activity, suggesting that binaural processing in the SOC is subject to GABA_B_R-mediated dynamic adaptation (128, 132, 133). In addition, as shown in mice and gerbils, GABA_B_Rs can be tonically activated by ambient GABA and regulate the excitability of LSO and MSO neurons *via* postsynaptic GIRK channels (131, 134). These effects are thought to protect principal cells from overexcitation caused by increased spontaneous activity entering the binaural nuclei around the onset of hearing (127). At this stage of auditory system development, GABA_B_Rs at glycinergic MNTB-LSO synapses also play important roles in mechanisms of long-term plasticity of inhibitory transmission. Before the onset of hearing, postsynaptic receptors mediate the depression (LTD) of MNTB-evoked inhibitory potentials (135, 136), whereas following the onset, GABA_B_R signaling is required to induce potentiation (LTP) of inhibitory postsynaptic currents (137). Based on these observations, it has been proposed that GABA_B_R-dependent plasticity underlies both the early elimination of redundant inhibitory synaptic connections in the LSO and their later stabilization and strengthening during subsequent postnatal development (137, 138).

Excitatory inputs of MNTB principal cells formed as giant axosomatic terminals, the calyces of Held (139–141) (**Figure 3A**), allow direct electrophysiological examination (142) and have therefore often been used to study the effects of GABA_B_Rs on neurotransmitter exocytosis. It has been found that activation of calyceal GABA_B_Rs receptors in rat brainstem slices blocks approximately 80% of glutamate release, by inhibiting presynaptic VGCCs through the action of G_βγ_ subunits (143–145). In addition, GABA_B_Rs can directly interfere with the vesicular cycle by reducing cAMP (30). The endogenous source of agonists for presynaptic GABA_B_Rs in MNTB remains unclear. It has been suggested that the receptors could be tonically activated by ambient GABA and subsequently help to maintain the relatively low basal probability of glutamate release and little short-term plasticity observed at the calyx of Held synapse *in vivo* (146–148). However, *in vivo* experiments with pharmacological modulation of GABA_B_R activity have not yet confirmed this hypothesis. No significant change in synaptic transmission in mice was observed during application of the GABA_B_R antagonist CGP54626, suggesting a low ambient GABA concentration (149). However, these experiments did not exclude the possibility that the application of antagonist also led to inhibition of presynaptic GABA_B_Rs at glycinergic synapses, thereby enhancing inhibitory transmission in the MNTB. This would in turn reduce the intensity of steady-state transmission at the calyx of Held synapse *via* pre- and postsynaptic glycine receptors, thus compensating for the effect of inhibition of calyceal GABA_B_Rs (150–154). Further *in vivo* experiments using glycine receptor antagonists would be needed to test this possibility.

### 4.3 Inferior colliculus

**Figure 4:**
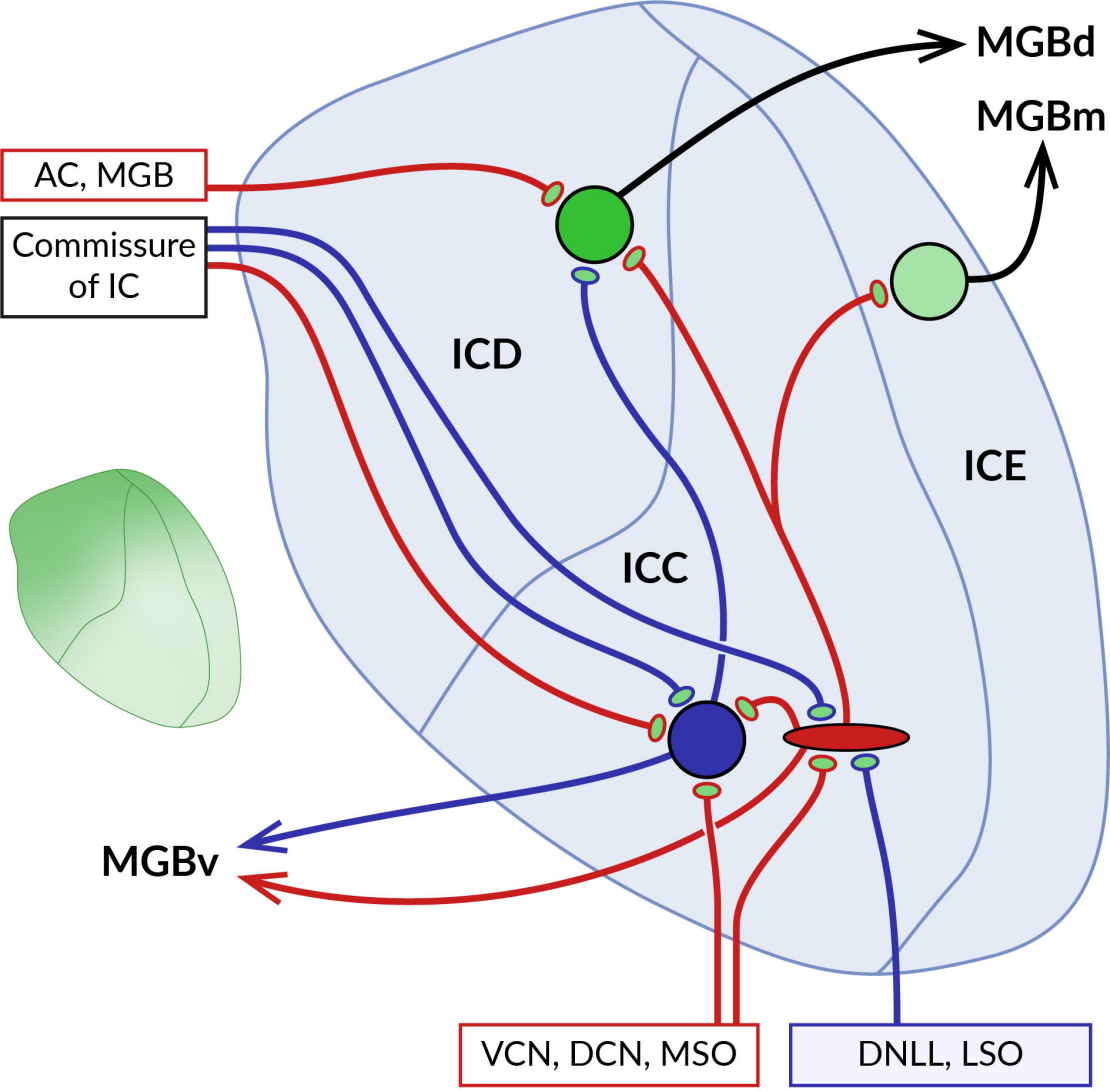
Localization of GABA_B_Rs at pre- and postsynaptic sites in the inferior colliculus (IC). Schematic representation of the main parts of the IC with their main afferent and efferent projections. The central core of the IC (ICC) is surrounded by the dorsal (ICD) and external cortex (ICE). Ascending excitatory (red) and inhibitory (blue) fibers from ventral and dorsal cochlear nuclei (VCN, DCN), medial superior olive (MSO), dorsal nucleus of the lateral lemniscus (DNLL), and lateral superior olive (LSO) contact large GABAergic and glutamatergic neurons in the ICC. Both types of ICC neurons project to cortical areas, predominantly to the ICD. The two ICs are interconnected by commissural fibers. Descending projections from the auditory cortex (AC) and medial geniculate body (MGB) terminate on ICD neurons. As the diagram shows, neurons in different parts of the IC preferentially project to distinct nuclei of the auditory thalamus, medial, dorsal and ventral MGB (MGBm, MGBd and MGBv). GABA_B_Rs in ICC show a preferential presynaptic localization, while cortical receptors occupy both pre- and postsynaptic locations. The inset on the left shows the gradient of the expression level of GABA_B_Rs in the IC, which reaches the highest values in the dorsomedial part of the IC and decreases ventrolaterally.

The inferior colliculus (IC) is a midbrain structure that connects the auditory regions of the hindbrain and forebrain. It consists of a central core surrounded by lateral, dorsal and rostral cortices (125, 155) (**Figure 4**). Ascending fibers from the auditory brainstem innervate mostly neurons in the central nucleus, while descending fibers from the auditory cortex and thalamus terminate mainly in the external and to a limited extent in the central part of the IC (125, 156). Efferent fibers from the IC ascend to the corresponding parts of the thalamic medial geniculate nucleus or give rise to caudally oriented projections to the lower brainstem (157–159). The two ICs are interconnected by commissural fibers abundantly formed as collaterals of projections of the central core neurons to the ipsilateral medial geniculate body (160). In addition, the IC receives input from numerous non-auditory areas (161). It has been proposed that the function of neurons in the central nucleus is to integrate ascending auditory information and generate *de novo* coding properties, while those in the IC cortex allow for the integration of multimodal information and the detection of novel stimulus features (162).

The distribution of GABA_B_Rs in the IC was studied in brain sections of rats and big brown bats using quantitative autoradiography and immunohistochemistry (13, 16, 17, 163). The receptors were found throughout the entire IC both in neuronal somata and in the neuropil, with the highest expression levels in the dorsomedial cortical part which was also characterized by an increased density of GABA_B_-positive cell bodies (see inset in **Figure 4**). Pharmacological modulation of receptor activity significantly affects the sound-evoked responses of IC neurons (164–168) by a mechanism that appears to differ between IC subdivisions. In the central nucleus, presynaptic GABA_B_Rs have been shown to control the release of glutamate and GABA from excitatory and inhibitory fibers that form the lemniscal ascending inputs for sound-driven signals (169–171). The latter GABA_B_ autoreceptors, by suppressing GABAergic inhibition, can also promote the induction of long-term potentiation of excitatory potentials in IC neurons (171). In these studies, no postsynaptic GABA_B_R responses were observed following baclofen application or stimulation of inhibitory fibers, suggesting that the receptors in the central nucleus act primarily at presynaptic sites. Conversely, in the dorsal cortex of the IC, GABA_B_R activation leads to both presynaptic and postsynaptic responses. The former is associated with a reduced release of glutamate and GABA from the endings of afferent fibers, similar to that in the central nucleus (172, 173). Postsynaptic receptor responses, elicited pharmacologically or by stimulation of commissural GABAergic input, involve changes in the activity of GIRK and VGCC effector channels and significantly affect the firing properties of neurons in this region (173). Thus, these data suggest that GABA_B_Rs in cortical parts of the IC may modulate sound-evoked neuronal activity through pre- and postsynaptic functions and may also directly mediate inhibitory synaptic transmission.

Endogenous GABA_B_R activation in the IC could be triggered by GABAergic projections originating from the dorsal and ventral nuclei of the lateral lemniscus, the superior paraolivary nucleus and the contralateral IC (155, 174–176), as well as by local inhibitory interneurons. GABAergic neurons account for 25% of all IC neurons (177) and represent a heterogeneous group composed of several subtypes, differing in synaptic organization and neuronal connections (178, 179). A well-studied subset of large GABAergic neurons receives convergent glutamatergic input from multiple sources, including the IC, lateral lemniscus, SOC, DCN, and auditory cortex (180–182), suggesting that these neurons are part of complex feedforward, feedback, or disinhibitory neuronal circuits in the auditory pathway. However, the precise mechanisms by which GABA_B_Rs expressed in IC neurons contribute to the function of these circuits have not yet been fully understood. Due to their wide distribution at presynaptic and postsynaptic sites and the relatively slow kinetics of their responses, GABA_B_Rs are thought to regulate overall neural sensitivity to sounds and to set the gain of signal processing in the IC (17, 165, 167, 168). This idea would be consistent with the observation that pharmacological receptor blockade increases the acoustic excitation of IC neurons but does not reduce the inhibition elicited by paired stimuli or increase the range of amplitude modulation rates that evoke phase locking (166, 167). Interestingly, antagonists acting at postsynaptic GABA_B_Rs were found to reduce response adaptation of specialized IC cortex neurons to repetitive sounds, a phenomenon known as stimulus-specific adaptation (SSA) (168). In this study, it was proposed that, unlike the deviant tone, the repetitive tone activates more inhibitory inputs releasing greater amounts of GABA, which then dampens the firing rate of the SSA neuron *via* its extrasynaptic GABA_B_Rs. While receptor block does not affect the onset of adaptation, suggesting that receptors are not involved in the generation of SSA (168), these observations provide evidence that GABA_B_Rs may serve as modulators of predictive coding in the IC.

## 5 The role of subcortical GABA_B_Rs in pathological conditions of the auditory system

Dysfunction of the GABA_B_R, an important modulator of cellular excitability, is expected to be part of the mechanisms underlying the neuronal hyperactivity that accompanies some of the known auditory pathologies. Examples of such conditions include noise-induced hearing loss caused by sudden acoustic trauma or prolonged exposure to noise levels above 85 dB (183). It is well documented that this overexposure can lead to death of the IHCs and OHCs, reduction of synaptic ribbons, death of spiral ganglion neurons, or degeneration of the auditory nerve, causing cochlear dysfunction and reduced sensory output (184–186). The peripheral deficit is then thought to trigger mechanisms of homeostatic plasticity in the central auditory pathway to regulate its gain and thereby compensate for the amount of neural activity from the cochlea (187–190). Although the mechanisms underlying neuronal gain modification are not fully understood, one possibility is a reduction in synaptic inhibition mediated by neurotransmitters such as GABA and glycine (191). Consistent with this assumption, studies in animal models of acoustic trauma indicated an imbalance between excitation and inhibition in the auditory system due to impaired GABAergic neurotransmission (192–196), including reduced GABA_B_R expression in the auditory brainstem (121, 122). The latter showed that neurons in the molecular and fusiform layers of the DCN exhibit reduced GABA_B_R density in mice after acoustic trauma, presumably as a result of receptor internalization due to its phosphorylation by protein kinase C gamma (121, 122). This is consistent with the increased excitability of these neurons in animals with noise-induced hearing loss (90, 197, 198). Thus, changes in GABA_B_ function may be part of the mechanisms underlying maladaptive plasticity in the DCN, which is known to lead to hyperactivity of DCN neurons and the development of tinnitus (199, 200). Changes in receptor expression or distribution could also occur in other regions of the subcortical auditory pathway, as suggested by trauma-induced changes in temporal and binaural processing or adaptive coding of sound stimuli, processes that require proper GABA_B_R function (165, 193, 201–204).

Tinnitus, phantom perception in the absence of sound stimuli, is another example of audiological conditions involving hyperactivity of neurons in the auditory pathway (see (90, 198, 205, 206) for reviews). It is generally ignited by hearing loss and very often by noise exposure (207) and, accordingly, animal models of tinnitus show reduced GABA/glycinergic inhibition in various regions of the auditory system (reviewed in (206, 208). Initial work focusing on the relationship between GABA_B_Rs and tinnitus explored the possibility of compensating for the loss of GABAergic inhibition by pharmacological activation of receptors with baclofen (165, 209). The results show that baclofen is a potent modulator of neuronal excitability in the ascending auditory pathway that dose-dependently reduces behavioral symptoms of chronic tinnitus in an animal model of acoustic trauma. Later work then indicated a closer link between the receptor and tinnitus by finding that intraperitoneal injection of sodium salicylate, which is known to elicit behavioral measures of tinnitus in animal studies (210), reduced levels of GABA_B1_ and GABA_B2_ subunits in the rat IC (211). These observations suggested that baclofen could represent a potentially effective agent in the treatment of tinnitus. However, this assumption failed to be demonstrated in a clinical study of this substance in patients with subjective tinnitus, in which no significant difference was found between the drug and placebo groups (212). Smith et al. (213) addressed the reasons for this failure and suggested that the efficacy of novel GABA_B_R agonists, which do not have the undesirable side effects of baclofen, should be investigated against tinnitus. It is also likely that tinnitus-associated changes in GABA_B_R expression occur only in selected cell subpopulations, as has been proposed, for example, for the inhibitory interneurons of the DCN, cartwheel cells (121). Therefore, more specific substances that selectively modulate the activity of GABA_B_Rs in specific cell types or compartments should be found for effective treatment. Interestingly, a variation in KCTD12 gene has been identified as a risk modifier in chronic tinnitus (214). As mentioned above, KCTD12 proteins exhibit multiple modulatory effects on GABA_B_R activity and are expressed in both the peripheral and central parts of the auditory system (11, 59). Their high expression in the stria vascularis suggests their possible involvement in the processes responsible for cochlear K+ transport necessary for the maintenance of the endocochlear potential (11, 85). Therefore, changes in KCTD12 action could contribute to sensorineural hearing loss due to impaired cochlear K+ homeostasis (215, 216) and impaired GABA_B_ function in the auditory pathway, leading to tinnitus. KCTD12 may thus represent a potential target in the therapy of hearing disorders, and it is therefore essential to understand both the mechanisms of its effects in the auditory system and its role in animal models of tinnitus.

## 6 Concluding remarks

The above data illustrate the involvement of GABA_B_Rs in mechanisms that are important for proper hearing function in mammals. These include both peripheral and central processes such as cochlear amplification, sound source localization, temporal processing, adaptive coding, or suppression of spontaneous activity. Because of their relatively slow kinetics, GABA_B_Rs are thought to primarily regulate the overall excitability of neurons and thus determine the gain in signal processing. The relatively broad involvement of receptors in auditory processing could then be due to their specific localization in neuronal circuits and the multitude of interacting proteins that serve as their effectors or through which other signaling pathways modulate GABA_B_R activity and thus neuronal responses. The presence of numerous modulation sites on the subunits of GABA_B_Rs and their effectors also provides an opportunity to influence receptor responses with pharmacological agents, for example those that restore GABA_B_ function in certain pathological conditions of the auditory system.

As discussed in Section 5, noise or salicylate-induced hearing loss may be accompanied by changes in central gain, and it is likely that attenuation of GABA_B_R signaling contributes to this. Similarly, GABA_B_Rs seem to be involved in mechanisms of age-related hearing loss, as suggested by the decline in their levels in the auditory pathway of aging rats (163, 217). By increasing the severity of the auditory deficit, reduced GABA_B_R expression could also indirectly promote pathophysiological processes in other, non-auditory parts of the brain. This assumption is based on the fact that the auditory pathway contains numerous inputs from other sensory systems (see also Sections 4.1 and 4. 3), through which it receives neuromodulation that affects sound processing, and through which it in turn elicits auditory-driven neural responses in non-auditory areas (see (161, 218, 219) for reviews and (220) for recent evidence). Growing evidence suggests a strong association between hearing impairment and cognitive decline, and age-related hearing loss is considered one of the greatest risk factors for the development of dementias, including Alzheimer’s disease (221, 222). The mechanisms linking these states are not entirely clear, but they appear to involve a cascade of events altering the activity of neural circuits in areas important for cognitive function, such as the hippocampus (223–226). Therefore, changes in the activity of auditory neurons due to GABA_B_R dysfunction may potentially play a role in these processes. Hearing loss could be widely treated with hearing aids or cochlear implants and is therefore considered a modifiable risk factor (225), but treatment targeting GABA_B_Rs in the auditory pathway could represent a pharmacological alternative. However, the mere systemic use of stimulators of GABA_B_R or its effectors could lead to contradictory effects, as pharmacological inhibition of GABA_B_Rs or their G-protein-dependent signaling has been shown to improve hippocampus-dependent memory and decision making, respectively (227–229). This suggests the need for new specific agents targeting GABA_B_Rs expressed in the auditory system. The current detailed knowledge of GABA_B_R structure supports advances in pharmacological methods, enabling the investigation of receptor function, as well as the development of new drugs, such as specific allosteric modulators and peptide-based inhibitors that target protein-protein interactions in the receptor complex (230, 231).

## Author contributions

RT wrote the paper with input from the other authors. All authors contributed to the article and approved the submitted version.

## References

[1] Kohl MM Paulsen O. The roles of GABAB receptors in cortical network activity. Adv Pharmacol (2010) 58:205–29. doi: 10.1016/S1054-3589(10)58009-8 20655484

[2] Heaney CF Kinney JW. Role of GABA(B) receptors in learning and memory and neurological disorders. Neurosci Biobehav Rev (2016) 63:1–28. doi: 10.1016/j.neubiorev.2016.01.007 26814961

[3] Malcangio M. GABA(B) receptors and pain. Neuropharmacology (2018) 136:102–5. doi: 10.1016/j.neuropharm.2017.05.012 28504122

[4] Kulik Á. Booker SA Vida I. Differential distribution and function of GABA(B)Rs in somato-dendritic and axonal compartments of principal cells and interneurons in cortical circuits. Neuropharmacology (2018) 136:80–91. doi: 10.1016/j.neuropharm.2017.10.018 29042314

[5] Gassmann M Bettler B. Regulation of neuronal GABAB receptor functions by subunit composition. Nat Rev Neurosci (2012) 13:380–94. doi: 10.1038/nrn3249 22595784

[6] Ge S Pradhan DA Ming G-L Song H. GABA sets the tempo for activity-dependent adult neurogenesis. Trends Neurosci (2007) 30:1–8. doi: 10.1016/j.tins.2006.11.001 17116335

[7] Bassetti D. Keeping the balance: GABA(B) receptors in the developing brain and beyond. Brain Sci (2022) 12:419. doi: 10.3390/brainsci12040419 35447949 PMC9031223

[8] Benke D. GABAB receptor trafficking and interacting proteins: targets for the development of highly specific therapeutic strategies to treat neurological disorders? Biochem Pharmacol (2013) 86:1525–30. doi: 10.1016/j.bcp.2013.09.016 24084431

[9] Filip M Frankowska M Sadakierska-Chudy A Suder A Szumiec L Mierzejewski P. GABAB receptors as a therapeutic strategy in substance use disorders: focus on positive allosteric modulators. Neuropharmacology (2015) 88:36–47. doi: 10.1016/j.neuropharm.2014.06.016 24971600

[10] Jacobson LH Vlachou S Slattery DA Li X Cryan JF. The gamma-aminobutyric acid B receptor in depression and reward. Biol Psychiatry (2018) 83:963–76. doi: 10.1016/j.biopsych.2018.02.006 29759132

[11] Resendes BL Kuo SF Robertson NG Giersch ABS Honrubia D Ohara O. Isolation from cochlea of a novel human intronless gene with predominant fetal expression. J Assoc Res Otolaryngol JARO (2004) 5:185–202. doi: 10.1007/s10162-003-4042-x 15357420 PMC2538407

[12] Juiz JM Albin RL Helfert RH Altschuler RA. Distribution of GABAA and GABAB binding sites in the cochlear nucleus of the Guinea pig. Brain Res (1994) 639:193–201. doi: 10.1016/0006-8993(94)91730-2 8205472

[13] Fubara BM Casseday JH Covey E Schwartz-Bloom RD. Distribution of GABAA, GABAB, and glycine receptors in the central auditory system of the big brown bat, Eptesicus fuscus. J Comp Neurol (1996) 369:83–92. doi: 10.1002/(SICI)1096-9861(19960520)369:1<83::AID-CNE6>3.0.CO;2-G 8723704

[14] Charles KJ Evans ML Robbins MJ Calver AR Leslie RA Pangalos MN. Comparative immunohistochemical localisation of GABA(B1a), GABA(B1b) and GABA(B2) subunits in rat brain, spinal cord and dorsal root ganglion. Neuroscience (2001) 106:447–67. doi: 10.1016/S0306-4522(01)00296-2 11591450

[15] Luján R Shigemoto R Kulik A Juiz JM. Localization of the GABAB receptor 1a/b subunit relative to glutamatergic synapses in the dorsal cochlear nucleus of the rat. J Comp Neurol (2004) 475:36–46. doi: 10.1002/cne.20160 15176083

[16] Jamal L Zhang H Finlayson PG Porter LA Zhang H. The level and distribution of the GABA(B)R2 receptor subunit in the rat's central auditory system. Neuroscience (2011) 6:243–56. doi: 10.1016/j.neuroscience.2011.02.050 21371537

[17] Jamal L Khan AN Butt S Patel CR Zhang H. The level and distribution of the GABABRI and GABABR2 receptor subunits in the rat's inferior colliculus. Front Neural Circuits (2012) 6:92. doi: 10.3389/fncir.2012.00092 23189044 PMC3506002

[18] Hilbig H Nowack S Boeckler K Bidmon H-J Zilles K. Characterization of neuronal subsets surrounded by perineuronal nets in the rhesus auditory brainstem. J Anat (2007) 210:507–17. doi: 10.1111/j.1469-7580.2007.00713.x PMC2375744 17451528

[19] Pin J-P Bettler B. Organization and functions of mGlu and GABA(B) receptor complexes. Nature (2016) 540:60–8. doi: 10.1038/nature20566 27905440

[20] Margeta-Mitrovic M Jan YN Jan LY. A trafficking checkpoint controls GABA(B) receptor heterodimerization. Neuron (2000) 27:97–106. doi: 10.1016/S0896-6273(00)00012-X 10939334

[21] Doly S Shirvani H Gäta G Meye FJ Emerit MB Enslen H. GABAB receptor cell-surface export is controlled by an endoplasmic reticulum gatekeeper. Mol Psychiatry (2016) 21:480–90. doi: 10.1038/mp.2015.72 PMC4828513 26033241

[22] Frangaj A Fan QR. Structural biology of GABA(B) receptor. Neuropharmacology (2018) 136:68–79. doi: 10.1016/j.neuropharm.2017.10.011 29031577 PMC5897222

[23] Fritzius T Bettler B. The organizing principle of GABA(B) receptor complexes: Physiological and pharmacological implications. Basic Clin Pharmacol Toxicol (2020) 126 Suppl:25–34. doi: 10.1111/bcpt.13241 31033219 PMC7317483

[24] Kniazeff J. The different aspects of the GABA(B) receptor allosteric modulation. Adv Pharmacol (San Diego Calif.) (2020) 88:83–113. doi: 10.1016/bs.apha.2020.02.003 32416873

[25] Whorton MR MacKinnon R. X-ray structure of the mamMalian GIRK2-βγ G-protein complex. Nature (2013) 498:190–7. doi: 10.1038/nature12241 PMC4654628 23739333

[26] Zamponi GW Currie KPM. Regulation of Ca(V)2 calcium channels by G protein coupled receptors. Biochim Biophys Acta (2013) 1828:1629–43. doi: 10.1016/j.bbamem.2012.10.004 PMC3556207 23063655

[27] Xu J Wojcik WJ. Gamma aminobutyric acid B receptor-mediated inhibition of adenylate cyclase in cultured cerebellar granule cells: blockade by islet-activating protein. J Pharmacol Exp Ther (1986) 239:568–73. 2430096

[28] Wu LG Saggau P. GABAB receptor-mediated presynaptic inhibition in Guinea-pig hippocampus is caused by reduction of presynaptic Ca2+ influx. J Physiol (1995) 485 Pt 3:649–57. doi: 10.1113/jphysiol.1995.sp020759 PMC1158034 7562607

[29] Lüscher C Jan LY Stoffel M Malenka RC Nicoll RA. G protein-coupled inwardly rectifying K+ channels (GIRKs) mediate postsynaptic but not presynaptic transmitter actions in hippocampal neurons. Neuron (1997) 19:687–95. doi: 10.1016/S0896-6273(00)80381-5 9331358

[30] Sakaba T Neher E. Direct modulation of synaptic vesicle priming by GABA(B) receptor activation at a glutamatergic synapse. Nature (2003) 424:775–8. doi: 10.1038/nature01859 12917685

[31] Pérez-Garci E Gassmann M Bettler B Larkum ME. The GABAB1b isoform mediates long-lasting inhibition of dendritic ca2+ Spikes in layer 5 somatosensory pyramidal neurons. Neuron (2006) 50:603–16. doi: 10.1016/j.neuron.2006.04.019 16701210

[32] Chalifoux JR Carter AG. GABAB receptor modulation of synaptic function. Curr Opin Neurobiol (2011) 21:339–44. doi: 10.1016/j.conb.2011.02.004 PMC3092847 21376567

[33] Zhang J Tan L Ren Y Liang J Lin R Feng Q. Presynaptic excitation *via* GABAB receptors in habenula cholinergic neurons regulates fear memory expression. Cell (2016) 166:716–28. doi: 10.1016/j.cell.2016.06.026 27426949

[34] Benke D Balakrishnan K Zemoura K. Regulation of cell surface GABA(B) receptors: contribution to synaptic plasticity in neurological diseases. Adv Pharmacol (San Diego Calif.) (2015) 73:41–70. doi: 10.1016/bs.apha.2014.11.002 25637437

[35] Terunuma M. Diversity of structure and function of GABA(B) receptors: a complexity of GABA(B)-mediated signaling. Proc Japan Academy. Ser B Phys Biol Sci (2018) 94:390–411. doi: 10.2183/pjab.94.026 PMC6374141 30541966

[36] Guetg N Abdel Aziz S Holbro N Turecek R Rose T Seddik R. NMDA receptor-dependent GABAB receptor internalization *via* CaMKII phosphorylation of serine 867 in GABAB1. Proc Natl Acad Sci United States America (2010) 107:13924–9. doi: 10.1073/pnas.1000909107 PMC2922270 20643921

[37] Pontier SM Lahaie N Ginham R St-Gelais F Bonin H Bell DJ. Coordinated action of NSF and PKC regulates GABABreceptor signaling efficacy. EMBO J (2006) 25:2698–709. doi: 10.1038/sj.emboj.7601157 PMC1500845 16724110

[38] Kuramoto N Wilkins ME Fairfax BP Revilla-Sanchez R Terunuma M Tamaki K. Phospho-dependent functional modulation of GABA(B) receptors by the metabolic sensor AMP-dependent protein kinase. Neuron (2007) 53:233–47. doi: 10.1016/j.neuron.2006.12.015 PMC2570046 17224405

[39] Couve A Thomas P Calver AR Hirst WD Pangalos MN Walsh FS. Cyclic AMP-dependent protein kinase phosphorylation facilitates GABA(B) receptor-effector coupling. Nat Neurosci (2002) 5:415–24. doi: 10.1038/nn833 11976702

[40] Vigot R Barbieri S Bräuner-Osborne H Turecek R Shigemoto R Zhang Y-P. Differential compartmentalization and distinct functions of GABAB receptor variants. Neuron (2006) 50:589–601. doi: 10.1016/j.neuron.2006.04.014 16701209 PMC3531664

[41] Biermann B Ivankova-Susankova K Bradaia A Abdel Aziz S Besseyrias V Kapfhammer JP. The sushi domains of GABAB receptors function as axonal targeting signals. J Neurosci (2010) 30:1385–94. doi: 10.1523/JNEUROSCI.3172-09.2010 PMC6633810 20107064

[42] Hannan S Wilkins ME Smart TG. Sushi domains confer distinct trafficking profiles on GABAB receptors. Proc Natl Acad Sci United States America (2012) 109:12171–6. doi: 10.1073/pnas.1201660109 PMC3409743 22778417

[43] Schwenk J Pérez-Garci E Schneider A Kollewe A Gauthier-Kemper A Fritzius T. Modular composition and dynamics of native GABAB receptors identified by high-resolution proteomics. Nat Neurosci (2016) 19:233–42. doi: 10.1038/nn.4198 26691831

[44] Dinamarca MC Raveh A Schneider A Fritzius T Früh S Rem PD. Complex formation of APP with GABA(B) receptors links axonal trafficking to amyloidogenic processing. Nat Commun (2019) 10:1331–1. doi: 10.1038/s41467-019-09164-3 PMC6430795 30902970

[45] Rice HC de Malmazet D Schreurs A Frere S Van Molle I Volkov AN. Secreted amyloid-β precursor protein functions as a GABA(B)R1a ligand to modulate synaptic transmission. Sci (New York N.Y.) (2019) 363:e4827. doi: 10.1126/science.aao4827 PMC6366617 30630900

[46] Rem PD Sereikaite V Fernández-Fernández D Reinartz S Ulrich D Fritzius T. Soluble amyloid-β precursor peptide does not regulate GABA(B) receptor activity. Elife (2023) 12:e82082. doi: 10.7554/eLife.82082 36688536 PMC9917443

[47] Couve A Kittler JT Uren JM Calver AR Pangalos MN Walsh FS. Association of GABA(B) receptors and members of the 14-3-3 family of signaling proteins. Mol Cell Neurosci (2001) 17:317–28. doi: 10.1006/mcne.2000.0938 11178869

[48] Hanack C Moroni M Lima WC Wende H Kirchner M Adelfinger L. GABA blocks pathological but not acute TRPV1 pain signals. Cell (2015) 160:759–70. doi: 10.1016/j.cell.2015.01.022 25679765

[49] Laffray S Bouali-Benazzouz R Papon M-A Favereaux A Jiang Y Holm T. Impairment of GABAB receptor dimer by endogenous 14-3-3ζ in chronic pain conditions. EMBO J (2012) 31:3239–51. doi: 10.1038/emboj.2012.161 PMC3411072 22692127

[50] Schwenk J Metz M Zolles G Turecek R Fritzius T Bildl W. Native GABABreceptors are heteromultimers with a family of auxiliary subunits. Nature (2010) 465:231–5. doi: 10.1038/nature08964 20400944

[51] Ivankova K Turecek R Fritzius T Seddik R Prezeau L Comps-Agrar L. Up-regulation of GABAB receptor signaling by constitutive assembly with the K+ channel tetramerization domain-containing protein 12 (KCTD12). J Biol Chem (2013) 288:24848–56. doi: 10.1074/jbc.M113.476770 PMC3750179 23843457

[52] Zuo H Glaaser I Zhao Y Kurinov I Mosyak L Wang H. Structural basis for auxiliary subunit KCTD16 regulation of the GABA(B) receptor. Proc Natl Acad Sci United States America (2019) 116:8370–9. doi: 10.1073/pnas.1903024116 PMC6486783 30971491

[53] Seddik R Jungblut SP Silander OK Rajalu M Fritzius T Besseyrias V. Opposite effects of KCTD subunit domains on GABABreceptor-mediated desensitization. J Biol Chem (2012) 287:39869–77. doi: 10.1074/jbc.M112.412767 PMC3501043 23035119

[54] Correale S Esposito C Pirone L Vitagliano L Di Gaetano S Pedone E. A biophysical characterization of the folded domains of KCTD12: insights into interaction with the GABAB2 receptor. J Mol recognition JMR (2013) 26:488–95. doi: 10.1002/jmr.2291 23996491

[55] Turecek R Schwenk J Fritzius T Ivankova K Zolles G Adelfinger L. Auxiliary GABAB receptor subunits uncouple G protein βγ subunits from effector channels to induce desensitization. Neuron (2014) 82:1032–44. doi: 10.1016/j.neuron.2014.04.015 24836506

[56] Zheng S Abreu N Levitz J Kruse AC. Structural basis for KCTD-mediated rapid desensitization of GABA(B) signalling. Nature (2019) 567:127–31. doi: 10.1038/s41586-019-0990-0 PMC6405316 30814734

[57] Adelfinger L Turecek R Ivankova K Jensen AA Moss SJ Gassmann M. GABAB receptor phosphorylation regulates KCTD12-induced K+ current desensitization. Biochem Pharmacol (2014) 91:369–79. doi: 10.1016/j.bcp.2014.07.013 PMC4402209 25065880

[58] Fritzius T Turecek R Seddik R Kobayashi H Tiao J Rem PD. KCTD hetero-oligomers confer unique kinetic properties on hippocampal GABAB receptor-induced K+ currents. J Neurosci (2017) 37:1162–75. doi: 10.1523/JNEUROSCI.2181-16.2016 PMC6596860 28003345

[59] Metz M Gassmann M Fakler B Schaeren-Wiemers N Bettler B. Distribution of the auxiliary GABAB receptor subunits KCTD8, 12, 12b, and 16 in the mouse brain. J Comp Neurol (2011) 519:1435–54. doi: 10.1002/cne.22610 21452234

[60] Ashmore J Avan P Brownell WE Dallos P Dierkes K Fettiplace R. The remarkable cochlear amplifier. Hearing Res (2010) 266:1–17. doi: 10.1016/j.heares.2010.05.001 PMC6366996 20541061

[61] Fettiplace R Transduction HC. Tuning, and synaptic transmission in the mamMalian cochlea. Compr Physiol (2017) 7:1197–227. doi: 10.1002/cphy.c160049 PMC5658794 28915323

[62] Reijntjes DOJ Pyott SJ. The afferent signaling complex: Regulation of type I spiral ganglion neuron responses in the auditory periphery. Hearing Res (2016) 336:1–16. doi: 10.1016/j.heares.2016.03.011 27018296

[63] Carricondo F Romero-Gómez B. The cochlear spiral ganglion neurons: the auditory portion of the VIII nerve. Anatomical Rec (Hoboken N.J. 2007) (2019) 302:463–71. doi: 10.1002/ar.23815 29659185

[64] Weisz CJC Glowatzki E Fuchs PA. Excitability of type II cochlear afferents. J Neurosci Off J Soc Neurosci (2014) 34:2365–73. doi: 10.1523/JNEUROSCI.3428-13.2014 PMC3913877 24501375

[65] Flores EN Duggan A Madathany T Hogan AK Márquez FG Kumar G. A non-canonical pathway from cochlea to brain signals tissue-damaging noise. Curr Biol CB (2015) 25:606–12. doi: 10.1016/j.cub.2015.01.009 PMC4348215 25639244

[66] Lin X Chen S Chen P. Activation of metabotropic GABAB receptors inhibited glutamate responses in spiral ganglion neurons of mice. Neuroreport (2000) 11:957–61. doi: 10.1097/00001756-200004070-00012 10790863

[67] Maison SF Casanova E Holstein GR Bettler B Liberman MC. Loss of GABAB receptors in cochlear neurons: threshold elevation suggests modulation of outer hair cell function by type II afferent fibers. J Assoc Res Otolaryngol JARO (2009) 10:50–63. doi: 10.1007/s10162-008-0138-7 18925381 PMC2644393

[68] Wedemeyer C Zorrilla de San Martín J Ballestero J Gómez-Casati ME Torbidoni AV Fuchs PA. Activation of presynaptic GABA(B(1a,2)) receptors inhibits synaptic transmission at mamMalian inhibitory cholinergic olivocochlear-hair cell synapses. J Neurosci Off J Soc Neurosci (2013) 33:15477–87. doi: 10.1523/JNEUROSCI.2554-13.2013 PMC3782624 24068816

[69] Shrestha BR Chia C Wu L Kujawa SG Liberman MC Goodrich LV. Sensory neuron diversity in the inner ear is shaped by activity. Cell (2018) 174:1229–46. doi: 10.1016/j.cell.2018.07.007 PMC6150604 30078709

[70] Sun S Babola T Pregernig G So KS Nguyen M Su S-SM. Hair cell mechanotransduction regulates spontaneous activity and spiral ganglion subtype specification in the auditory system. Cell (2018) 174:1247–1263.e15. doi: 10.1016/j.cell.2018.07.008 30078710 PMC6429032

[71] Ruel J Chabbert C Nouvian R Bendris R Eybalin M Leger CL. Salicylate enables cochlear arachidonic-acid-sensitive NMDA receptor responses. J Neurosci Off J Soc Neurosci (2008) 28:7313–23. doi: 10.1523/JNEUROSCI.5335-07.2008 PMC6670386 18632935

[72] Petitpré C Bourien J Wu H Diuba A Puel J-L Lallemend F. Genetic and functional diversity of primary auditory afferents. Curr Opin Physiol (2020) 18:85–94. doi: 10.1016/j.cophys.2020.09.011

[73] Ramakrishna Y Manca M Glowatzki E Sadeghi SG. Cholinergic modulation of membrane properties of calyx terminals in the vestibular periphery. Neuroscience (2021) 452:98–110. doi: 10.1016/j.neuroscience.2020.10.035 33197502 PMC8054478

[74] Lv P Kim HJ Lee J-H Sihn C-R Fathabad Gharaie S Mousavi-Nik A. Genetic, cellular, and functional evidence for Ca2+ inflow through Cav1.2 and Cav1.3 channels in murine spiral ganglion neurons. J Neurosci Off J Soc Neurosci (2014) 34:7383–93. doi: 10.1523/JNEUROSCI.5416-13.2014 PMC4028507 24849370

[75] Pyott SJ Duncan RK. BK channels in the vertebrate inner ear. Int Rev Neurobiol (2016) 128:369–99. doi: 10.1016/bs.irn.2016.03.016 27238269

[76] Petitpré C Wu H Sharma A Tokarska A Fontanet P Wang Y. Neuronal heterogeneity and stereotyped connectivity in the auditory afferent system. Nat Commun (2018) 9:3691–1. doi: 10.1038/s41467-018-06033-3 PMC6135759 30209249

[77] Guinan JJ Jr. Olivocochlear efferents: Their action, effects, measurement and uses, and the impact of the new conception of cochlear mechanical responses. Hear Res (2018) 362:38–47. doi: 10.1016/j.heares.2017.12.012 29291948 PMC5911200

[78] Wedemeyer C Vattino LG Moglie MJ Ballestero J Maison SF Di Guilmi MN. A gain-of-function mutation in the α9 nicotinic acetylcholine receptor alters medial olivocochlear efferent short-term synaptic plasticity. J Neurosci Off J Soc Neurosci (2018) 38:3939–54. doi: 10.1523/JNEUROSCI.2528-17.2018 PMC5907056 29572431

[79] Nouvian R Eybalin M Puel J-L. Cochlear efferents in developing adult and pathological conditions. Cell Tissue Res (2015) 361:301–9. doi: 10.1007/s00441-015-2158-z 25810366

[80] Katz E Elgoyhen AB. Short-term plasticity and modulation of synaptic transmission at mamMalian inhibitory cholinergic olivocochlear synapses. Front Syst Neurosci (2014) 8:224–4. doi: 10.3389/fnsys.2014.00224 PMC4251319 25520631

[81] Maison SF Adams JC Liberman MC. Olivocochlear innervation in the mouse: immunocytochemical maps, crossed versus uncrossed contributions, and transmitter colocalization. J Comp Neurol (2003) 455:406–16. doi: 10.1002/cne.10490 PMC1805785 12483691

[82] Liberman MC Brown MC. Physiology and anatomy of single olivocochlear neurons in the cat. Hearing Res (1986) 24:17–36. doi: 10.1016/0378-5955(86)90003-1 3759672

[83] Mapelli L Rossi P Nieus T D'Angelo E. Tonic activation of GABAB receptors reduces release probability at inhibitory connections in the cerebellar glomerulus. J Neurophysiol (2009) 101:3089–99. doi: 10.1152/jn.91190.2008 19339456

[84] Vattino LG Wedemeyer C Elgoyhen AB Katz E. Functional postnatal maturation of the medial olivocochlear efferent-outer hair cell synapse. J Neurosci Off J Soc Neurosci (2020) 40:4842–57. doi: 10.1523/JNEUROSCI.2409-19.2020 PMC7326359 32430293

[85] Furness DN. Forgotten fibrocytes: A neglected, supporting cell type of the cochlea with the potential to be an alternative therapeutic target in hearing loss. Front Cell Neurosci (2019) 13:532–2. doi: 10.3389/fncel.2019.00532 PMC6908467 31866825

[86] Gale D.J.J. J.E. Cochlear supporting cells. In: The oxford handbook of auditory science: the ear. Oxford: Oxford University Press (2010). p. 307–27.

[87] Oertel D Young ED. What's a cerebellar circuit doing in the auditory system? Trends Neurosci (2004) 27:104–10. doi: 10.1016/j.tins.2003.12.001 15102490

[88] Rubio ME. Microcircuits of the ventral cochlear nucleus. In: Oliver DL Cant NB Fay RR Popper AN, editors. The Mammalian Auditory Pathways: Synaptic Organization and Microcircuits. Cham: Springer International Publishing (2018). p. 41–71.

[89] Trussell LO Oertel D. Microcircuits of the dorsal cochlear nucleus. In: Oliver DL Cant NB Fay RR Popper AN, editors. The Mammalian Auditory Pathways: Synaptic Organization and Microcircuits. Cham: Springer International Publishing (2018). p. 73–99.

[90] Shore SE Wu C. Mechanisms of noise-induced tinnitus: insights from cellular studies. Neuron (2019) 103:8–20. doi: 10.1016/j.neuron.2019.05.008 31271756 PMC6613804

[91] Shore SE. Multisensory integration in the dorsal cochlear nucleus: unit responses to acoustic and trigeminal ganglion stimulation. Eur J Neurosci (2005) 21:3334–48. doi: 10.1111/j.1460-9568.2005.04142.x 16026471

[92] Caspary DM Rybak LP Faingold CL. Baclofen reduces tone-evoked activity of cochlear nucleus neurons. Hearing Res (1984) 13:113–22. doi: 10.1016/0378-5955(84)90102-3 6325378

[93] Irie T Ohmori H. Presynaptic GABA(B) receptors modulate synaptic facilitation and depression at distinct synapses in fusiform cells of mouse dorsal cochlear nucleus. Biochem Biophys Res Commun (2008) 367:503–8. doi: 10.1016/j.bbrc.2008.01.001 18190780

[94] Cao X-J Oertel D. Auditory nerve fibers excite targets through synapses that vary in convergence, strength, and short-term plasticity. J Neurophysiol (2010) 104:2308–20. doi: 10.1152/jn.00451.2010 PMC3350034 20739600

[95] Wang Y Wang M Xie R. D-stellate neurons of the ventral cochlear nucleus decrease in auditory nerve-evoked activity during age-related hearing loss. Brain Sci (2019) 9:302. doi: 10.3390/brainsci9110302 31683609 PMC6896102

[96] Berger C Meyer EMM Ammer JJ Felmy F. Large somatic synapses on neurons in the ventral lateral lemniscus work in pairs. J Neurosci Off J Soc Neurosci (2014) 34:3237–46. doi: 10.1523/JNEUROSCI.3664-13.2014 PMC6795299 24573282

[97] Ngodup T Romero GE Trussell LO. Identification of an inhibitory neuron subtype, the L-stellate cell of the cochlear nucleus. eLife (2020) 9:e54350. doi: 10.7554/eLife.54350 33141020 PMC7744103

[98] Ryugo DK Parks TN. Primary innervation of the avian and mamMalian cochlear nucleus. Brain Res Bull (2003) 60:435–56. doi: 10.1016/S0361-9230(03)00049-2 12787866

[99] Chanda S Xu-Friedman MA. Neuromodulation by GABA converts a relay into a coincidence detector. J Neurophysiol (2010) 104:2063–74. doi: 10.1152/jn.00474.2010 PMC2957460 20702743

[100] Chanda S Oh S Xu-Friedman MA. Calcium imaging of auditory nerve fiber terminals in the cochlear nucleus. J Neurosci Methods (2011) 195:24–9. doi: 10.1016/j.jneumeth.2010.11.008 PMC3019277 21108967

[101] Zhuang X Wong NF Sun W Xu-Friedman MA. Mechanisms and functional consequences of presynaptic homeostatic plasticity at auditory nerve synapses. J Neurosci Off J Soc Neurosci (2020) 40:6896–909. doi: 10.1523/JNEUROSCI.1175-19.2020 PMC7470918 32747441

[102] Joris PX Carney LH Smith PH Yin TC. Enhancement of neural synchronization in the anteroventral cochlear nucleus. I. Responses to tones at the characteristic frequency. J Neurophysiol (1994) 71:1022–36. doi: 10.1152/jn.1994.71.3.1022 8201399

[103] Xu-Friedman MA Regehr WG. Dynamic-clamp analysis of the effects of convergence on spike timing. II. Few synaptic inputs. J Neurophysiol (2005) 94:2526–34. doi: 10.1152/jn.01308.2004 16160093

[104] Doucet JR Ryugo DK. Structural and functional classes of multipolar cells in the ventral cochlear nucleus. anatomical Rec Part A Discoveries molecular cellular evolutionary Biol (2006) 288:331–44. doi: 10.1002/ar.a.20294 PMC2566305 16550550

[105] Needham K Paolini AG. The commissural pathway and cochlear nucleus bushy neurons: an in *vivo* intracellular investigation. Brain Res (2007) 1134:113–21. doi: 10.1016/j.brainres.2006.11.058 17174943

[106] Lim R Alvarez FJ Walmsley B. GABA mediates presynaptic inhibition at glycinergic synapses in a rat auditory brainstem nucleus. J Physiol (2000) 525 Pt 2:447–59. doi: 10.1111/j.1469-7793.2000.t01-1-00447.x PMC2269953 10835046

[107] Muniak MA Ryugo DK. Tonotopic organization of vertical cells in the dorsal cochlear nucleus of the CBA/J mouse. J Comp Neurol (2014) 522:937–49. doi: 10.1002/cne.23454 PMC3947158 23982998

[108] Benson TE Brown MC. Postsynaptic targets of type II auditory nerve fibers in the cochlear nucleus. J Assoc Res Otolaryngol JARO (2004) 5:111–25. doi: 10.1007/s10162-003-4012-3 PMC2538406 15357415

[109] Haenggeli C-A Pongstaporn T Doucet JR Ryugo DK. Projections from the spinal trigeminal nucleus to the cochlear nucleus in the rat. J Comp Neurol (2005) 484:191–205. doi: 10.1002/cne.20466 15736230

[110] Zhou J Shore S. Convergence of spinal trigeminal and cochlear nucleus projections in the inferior colliculus of the Guinea pig. J Comp Neurol (2006) 495:100–12. doi: 10.1002/cne.20863 16432905

[111] Evans EF Zhao W. Varieties of inhibition in the processing and control of processing in the mamMalian cochlear nucleus. Prog Brain Res (1993) 97:117–26. doi: 10.1016/S0079-6123(08)62269-4 7901869

[112] Harasztosi C Forsythe ID Szûcs G Stanfield PR Rusznák Z. Possible modulatory role of voltage-activated Ca(2+) currents determining the membrane properties of isolated pyramidal neurones of the rat dorsal cochlear nucleus. Brain Res (1999) 839:109–19. doi: 10.1016/S0006-8993(99)01723-0 10482805

[113] Shore SE Moore JK. Sources of input to the cochlear granule cell region in the Guinea pig. Hearing Res (1998) 116:33–42. doi: 10.1016/S0378-5955(97)00207-4 9508026

[114] Schofield BR Cant NB. Descending auditory pathways: projections from the inferior colliculus contact superior olivary cells that project bilaterally to the cochlear nuclei. J Comp Neurol (1999) 409:210–23. doi: 10.1002/(SICI)1096-9861(19990628)409:2<210::AID-CNE3>3.0.CO;2-A 10379915

[115] Thompson AM Schofield BR. Afferent projections of the superior olivary complex. Microscopy Res technique (2000) 51:330–54. doi: 10.1002/1097-0029(20001115)51:4<330::AID-JEMT4>3.0.CO;2-X 11071718

[116] Mancilla JG Manis PB. Two distinct types of inhibition mediated by cartwheel cells in the dorsal cochlear nucleus. J Neurophysiol (2009) 102:1287–95. doi: 10.1152/jn.91272.2008 PMC2724367 19474167

[117] Davis KA Young ED. Granule cell activation of complex-spiking neurons in dorsal cochlear nucleus. J Neurosci Off J Soc Neurosci (1997) 17:6798–806. doi: 10.1523/JNEUROSCI.17-17-06798.1997 PMC6573148 9254690

[118] Wu C Shore SE. Inhibitory interneurons in a brainstem circuit adjust their inhibitory motifs to process multimodal input. J Physiol (2021) 599:631–45. doi: 10.1113/JP280741 PMC7855092 33103245

[119] Golding NL Oertel D. Physiological identification of the targets of cartwheel cells in the dorsal cochlear nucleus. J Neurophysiol (1997) 78:248–60. doi: 10.1152/jn.1997.78.1.248 9242277

[120] Roberts MT Trussell LO. Molecular layer inhibitory interneurons provide feedforward and lateral inhibition in the dorsal cochlear nucleus. J Neurophysiol (2010) 104:2462–73. doi: 10.1152/jn.00312.2010 PMC2997026 20719922

[121] Kou ZZ Qu J Zhang DL Li H Li YQ. Noise-induced hearing loss is correlated with alterations in the expression of GABAB receptors and PKC gamma in the murine cochlear nucleus complex. Front Neuroanat (2013) 7:25. doi: 10.3389/fnana.2013.00025 23908607 PMC3726868

[122] Qu J Liao YH Kou ZZ Wei YY Huang J Chen J. Puerarin alleviates noise-induced hearing loss *via* affecting PKCγ and GABAB receptor expression. J Neurol Sci (2015) 349:110–6. doi: 10.1016/j.jns.2014.12.038 25592416

[123] Kuo SP Trussell LO. Spontaneous spiking and synaptic depression underlie noradrenergic control of feed-forward inhibition. Neuron (2011) 71:306–18. doi: 10.1016/j.neuron.2011.05.039 PMC3153142 21791289

[124] Grothe B Pecka M McAlpine D. Mechanisms of sound localization in mammals. Physiol Rev (2010) 90:983–1012. doi: 10.1152/physrev.00026.2009 20664077

[125] Cant NB Oliver DL. Overview of auditory projection pathways and intrinsic microcircuits. In: Oliver DL Cant NB Fay RR Popper AN, editors. The Mammalian Auditory Pathways: Synaptic Organization and Microcircuits. Cham: Springer International Publishing (2018). p. 7–39.

[126] Joris PX van der Heijden M. Early binaural hearing: the comparison of temporal differences at the two ears. Annu Rev Neurosci (2019) 42:433–57. doi: 10.1146/annurev-neuro-080317-061925 31018099

[127] Grothe B Koch U. Dynamics of binaural processing in the mamMalian sound localization pathway–the role of GABA(B) receptors. Hear Res (2011) 279:43–50. doi: 10.1016/j.heares.2011.03.013 21447375

[128] Grothe B Pecka M. The natural history of sound localization in mammals–a story of neuronal inhibition. Front Neural Circuits (2014) 8:116. doi: 10.3389/fncir.2014.00116 25324726 PMC4181121

[129] Magnusson AK Park TJ Pecka M Grothe B Koch U. Retrograde GABA signaling adjusts sound localization by balancing excitation and inhibition in the brainstem. Neuron (2008) 59:125–37. doi: 10.1016/j.neuron.2008.05.011 18614034

[130] Stange A Myoga MH Lingner A Ford MC Alexandrova O Felmy F. Adaptation in sound localization: from GABA(B) receptor-mediated synaptic modulation to perception. Nat Neurosci (2013) 16:1840–7. doi: 10.1038/nn.3548 24141311

[131] Fischer AU Müller NIC Deller T Del Turco D Fisch JO Griesemer D. GABA is a modulator, rather than a classical transmitter, in the medial nucleus of the trapezoid body-lateral superior olive sound localization circuit. J Physiol (2019) 597:2269–95. doi: 10.1113/JP277566 PMC6462465 30776090

[132] Fischl MJ Combs TD Klug A Grothe B Burger RM. Modulation of synaptic input by GABAB receptors improves coincidence detection for computation of sound location. J Physiol (2012) 590:3047–66. doi: 10.1113/jphysiol.2011.226233 PMC3406390 22473782

[133] Lingner A Pecka M Leibold C Grothe B. A novel concept for dynamic adjustment of auditory space. Sci Rep (2018) 8:8335. doi: 10.1038/s41598-018-26690-0 29844516 PMC5974081

[134] Hassfurth B Grothe B Koch U. The mamMalian interaural time difference detection circuit is differentially controlled by GABAB receptors during development. J Neurosci (2010) 30:9715–27. doi: 10.1523/JNEUROSCI.1552-10.2010 PMC6632825 20660254

[135] Kotak VC DiMattina C Sanes DH. GABA(B) and Trk receptor signaling mediates long-lasting inhibitory synaptic depression. J Neurophysiol (2001) 86:536–40. doi: 10.1152/jn.2001.86.1.536 11431532

[136] Chang EH Kotak VC Sanes DH. Long-term depression of synaptic inhibition is expressed postsynaptically in the developing auditory system. J Neurophysiol (2003) 90:1479–88. doi: 10.1152/jn.00386.2003 12761279

[137] Kotak VC Sanes DH. Developmental expression of inhibitory synaptic long-term potentiation in the lateral superior olive. Front Neural Circuits (2014) 8:67. doi: 10.3389/fncir.2014.00067 24994969 PMC4063273

[138] Takesian AE Kotak VC Sanes DH. Developmental hearing loss disrupts synaptic inhibition: implications for auditory processing. Future Neurol (2009) 4:331–49. doi: 10.2217/fnl.09.5 PMC2716048 20161214

[139] Borst JG Soria van Hoeve J. The calyx of Held synapse: from model synapse to auditory relay. Annu Rev Physiol (2012) 74:199–224. doi: 10.1146/annurev-physiol-020911-153236 22035348

[140] Schneggenburger R Forsythe ID. The calyx of held. Cell Tissue Res (2006) 326:311–37. doi: 10.1007/s00441-006-0272-7 16896951

[141] Joris PX Trussell LO. The calyx of held: A hypothesis on the need for reliable timing in an intensity-difference encoder. Neuron (2018) 100:534–49. doi: 10.1016/j.neuron.2018.10.026 PMC6263157 30408442

[142] Forsythe ID. Direct patch recording from identified presynaptic terminals mediating glutamatergic EPSCs in the rat CNS, in vitro. J Physiol (1994) 479(Pt 3):381–7. doi: 10.1113/jphysiol.1994.sp020303 PMC1155757 7837096

[143] Isaacson JS. GABAB receptor-mediated modulation of presynaptic currents and excitatory transmission at a fast central synapse. J Neurophysiol (1998) 80:1571–6. doi: 10.1152/jn.1998.80.3.1571 9744963

[144] Takahashi T Kajikawa Y Tsujimoto T. G-Protein-coupled modulation of presynaptic calcium currents and transmitter release by a GABAB receptor. J Neurosci (1998) 18:3138–46. doi: 10.1523/JNEUROSCI.18-09-03138.1998 PMC6792650 9547222

[145] Kajikawa Y Saitoh N Takahashi T. GTP-binding protein beta gamma subunits mediate presynaptic calcium current inhibition by GABA(B) receptor. Proc Natl Acad Sci U.S.A. (2001) 98:8054–8. doi: 10.1073/pnas.141031298 PMC35466 11416164

[146] Taschenberger H von Gersdorff H. Fine-tuning an auditory synapse for speed and fidelity: developmental changes in presynaptic waveform, EPSC kinetics, and synaptic plasticity. J Neurosci (2000) 20:9162–73. doi: 10.1523/JNEUROSCI.20-24-09162.2000 PMC6773022 11124994

[147] Sonntag M Englitz B Typlt M Rübsamen R. The calyx of Held develops adult-like dynamics and reliability by hearing onset in the mouse in vivo. J Neurosci (2011) 31:6699–709. doi: 10.1523/JNEUROSCI.0575-11.2011 PMC6632847 21543599

[148] Lorteije JA Rusu SI Kushmerick C Borst JG. Reliability and precision of the mouse calyx of Held synapse. J Neurosci (2009) 29:13770–84. doi: 10.1523/JNEUROSCI.3285-09.2009 PMC6666705 19889989

[149] Wang T Rusu SI Hruskova B Turecek R Borst JG. Modulation of synaptic depression of the calyx of Held synapse by GABA(B) receptors and spontaneous activity. J Physiol (2013) 591:4877–94. doi: 10.1113/jphysiol.2013.256875 PMC3800460 23940376

[150] Awatramani GB Turecek R Trussell LO. Inhibitory control at a synaptic relay. J Neurosci (2004) 24:2643–7. doi: 10.1523/JNEUROSCI.5144-03.2004 PMC6729505 15028756

[151] Turecek R Trussell LO. Presynaptic glycine receptors enhance transmitter release at a mamMalian central synapse. Nature (2001) 411:587–90. doi: 10.1038/35079084 11385573

[152] Hruskova B Trojanova J Kulik A Kralikova M Pysanenko K Bures Z. Differential distribution of glycine receptor subtypes at the rat calyx of Held synapse. J Neurosci (2012) 32:17012–24. doi: 10.1523/JNEUROSCI.1547-12.2012 PMC3531607 23175852

[153] Trojanova J Kulik A Janacek J Kralikova M Syka J Turecek R. Distribution of glycine receptors on the surface of the mature calyx of Held nerve terminal. Front Neural Circuits (2014) 8:120. doi: 10.3389/fncir.2014.00120 25339867 PMC4186306

[154] Hruskova B Trojanova J Kralikova M Melichar A Suchankova S Bartosova J. Cochlear ablation in neonatal rats disrupts inhibitory transmission in the medial nucleus of the trapezoid body. Neurosci Lett (2019) 699:145–50. doi: 10.1016/j.neulet.2019.01.058 30742935

[155] Saldaña E Merchán MA. Intrinsic and commissural connections of the inferior colliculus. In: Winer JA Schreiner CE, editors. The inferior colliculus. New York, NY: Springer New York (2005). p. 155–81.

[156] Malmierca MS Anderson LA Antunes FM. The cortical modulation of stimulus-specific adaptation in the auditory midbrain and thalamus: a potential neuronal correlate for predictive coding. Front Syst Neurosci (2015) 9:19. doi: 10.3389/fnsys.2015.00019 25805974 PMC4353371

[157] Caicedo A Herbert H. Topography of descending projections from the inferior colliculus to auditory brainstem nuclei in the rat. J Comp Neurol (1993) 328:377–92. doi: 10.1002/cne.903280305 7680052

[158] Mellott JG Foster NL Ohl AP Schofield BR. Excitatory and inhibitory projections in parallel pathways from the inferior colliculus to the auditory thalamus. Front Neuroanat (2014) 8:124. doi: 10.3389/fnana.2014.00124 25414646 PMC4220731

[159] Malmierca MS Le Beau FE Rees A. The topographical organization of descending projections from the central nucleus of the inferior colliculus in Guinea pig. Hearing Res (1996) 93:167–80. doi: 10.1016/0378-5955(95)00227-8 8735077

[160] González Hernández TH Meyer G Ferres-Torres R. The commissural interconnections of the inferior colliculus in the albino mouse. Brain Res (1986) 368:268–76. doi: 10.1016/0006-8993(86)90571-8 2421840

[161] Gruters KG Groh JM. Sounds and beyond: multisensory and other non-auditory signals in the inferior colliculus. Front Neural circuits (2012) 6:96. doi: 10.3389/fncir.2012.00096 23248584 PMC3518932

[162] Ito T Malmierca MS. Neurons, connections, and microcircuits of the inferior colliculus. In: Oliver DL Cant NB Fay RR Popper AN, editors. The Mammalian Auditory Pathways: Synaptic Organization and Microcircuits. Cham: Springer International Publishing (2018). p. 127–67.

[163] Milbrandt JC Albin RL Caspary DMC. Age-related decrease in GABAB receptor binding in the Fischer 344 rat i inferior colliculus. Neurobiol Aging (1994) 15:699–703. doi: 10.1016/0197-4580(94)90051-5 7891824

[164] Faingold CL Gehlbach G Caspary DM. On the role of GABA as an inhibitory neurotransmitter in inferior colliculus neurons: iontophoretic studies. Brain Res (1989) 500:302–12. doi: 10.1016/0006-8993(89)90326-0 2605499

[165] Szczepaniak WS Møller AR. Effects of (-)-baclofen, clonazepam, and diazepam on tone exposure-induced hyperexcitability of the inferior colliculus in the rat: possible therapeutic implications for pharmacological management of tinnitus and hyperacusis. Hear Res (1996) 97:46–53. doi: 10.1016/S0378-5955(96)80006-2 8844185

[166] Vaughn MD Pozza MF Lingenhöhl K. Excitatory acoustic responses in the inferior colliculus of the rat are increased by GABAB receptor blockade. Neuropharmacology (1996) 35:1761–7. doi: 10.1016/S0028-3908(96)00143-8 9076755

[167] Burger RM Pollak GD. Analysis of the role of inhibition in shaping responses to sinusoidally amplitude-modulated signals in the inferior colliculus. J Neurophysiol (1998) 80:1686–701. doi: 10.1152/jn.1998.80.4.1686 9772232

[168] Ayala YA Malmierca MS. The effect of inhibition on stimulus-specific adaptation in the inferior colliculus. Brain structure Funct (2018) 223:1391–407. doi: 10.1007/s00429-017-1546-4 29143124

[169] Ma CL Kelly JB Wu SH. Presynaptic modulation of GABAergic inhibition by GABA(B) receptors in the rat's inferior colliculus. Neuroscience (2002) 114:207–15. doi: 10.1016/S0306-4522(02)00130-6 12207966

[170] Sun H Ma CL Kelly JB Wu SH. GABAB receptor-mediated presynaptic inhibition of glutamatergic transmission in the inferior colliculus. Neurosci Lett (2006) 399:151–6. doi: 10.1016/j.neulet.2006.01.049 16513264

[171] Zhang Y Wu SH. Long-term potentiation in the inferior colliculus studied in rat brain slice. Hearing Res (2000) 147:92–103. doi: 10.1016/S0378-5955(00)00123-4 10962176

[172] Li Y Evans MS Faingold CL. Synaptic response patterns of neurons in the cortex of rat inferior colliculus. Hearing Res (1999) 137:15–28. doi: 10.1016/S0378-5955(99)00129-X 10545630

[173] Sun H Wu SH. The physiological role of pre- and postsynaptic GABA(B) receptors in membrane excitability and synaptic transmission of neurons in the rat's dorsal cortex of the inferior colliculus. Neuroscience (2009) 160:198–211. doi: 10.1016/j.neuroscience.2009.02.011 19409201

[174] Shneiderman A Oliver DL. EM autoradiographic study of the projections from the dorsal nucleus of the lateral lemniscus: a possible source of inhibitory inputs to the inferior colliculus. J Comp Neurol (1989) 286:28–47. doi: 10.1002/cne.902860103 2768557

[175] González-Hernández T Mantolán-Sarmiento B González-González B Pérez-González H. Sources of GABAergic input to the inferior colliculus of the rat. J Comp Neurol (1996) 372:309–26. doi: 10.1002/(SICI)1096-9861(19960819)372:2<309::AID-CNE11>3.0.CO;2-E 8863133

[176] Kulesza RJ Jr. Berrebi AS. Superior paraolivary nucleus of the rat is a GABAergic nucleus. J Assoc Res Otolaryngol JARO (2000) 1:255–69. doi: 10.1007/s101620010054 PMC2957197 11547806

[177] Merchán M Aguilar LA Lopez-Poveda EA Malmierca MS. The inferior colliculus of the rat: Quantitative immunocytochemical study of GABA and glycine. Neuroscience (2005) 136:907–25. doi: 10.1016/j.neuroscience.2004.12.030 16344160

[178] Ito T Bishop DC Oliver DL. Two classes of GABAergic neurons in the inferior colliculus. J Neurosci (2009) 29:13860–9. doi: 10.1523/JNEUROSCI.3454-09.2009 PMC2814801 19889997

[179] Beebe NL Young JW Mellott JG Schofield BR. Extracellular molecular markers and soma size of inhibitory neurons: Evidence for four subtypes of GABAergic cells in the inferior colliculus. J Neurosci (2016) 36:3988–99. doi: 10.1523/JNEUROSCI.0217-16.2016 PMC4821910 27053206

[180] Ito T Oliver DL. Local and commissural IC neurons make axosomatic inputs on large GABAergic tectothalamic neurons. J Comp Neurol (2014) 522:3539–54. doi: 10.1002/cne.23623 PMC4139440 24796971

[181] Ito T Hioki H Sohn J Okamoto S Kaneko T Iino S. Convergence of lemniscal and local excitatory inputs on large GABAergic tectothalamic neurons. J Comp Neurol (2015) 523:2277–96. doi: 10.1002/cne.23789 PMC5446767 25879870

[182] Chen C Cheng M Ito T Song S. Neuronal organization in the inferior colliculus revisited with cell-type-dependent monosynaptic tracing. J Neurosci Off J Soc Neurosci (2018) 38:3318–32. doi: 10.1523/JNEUROSCI.2173-17.2018 PMC6596054 29483283

[183] Levihaiem A. Noise induced hearing loss: the impact of acoustic trauma on the ear. Sci J Lander Coll Arts Sci (2015) 9.

[184] Baizer JS Wong KM Manohar S Hayes SH Ding D Dingman R. Effects of acoustic trauma on the auditory system of the rat: The role of microglia. Neuroscience (2015) 303:299–311. doi: 10.1016/j.neuroscience.2015.07.004 26162240 PMC4532607

[185] Kujawa SG Liberman MC. Adding insult to injury: cochlear nerve degeneration after "temporary" noise-induced hearing loss. J Neurosci (2009) 29:14077–85. doi: 10.1523/JNEUROSCI.2845-09.2009 PMC2812055 19906956

[186] Mao H Chen Y. Noise-induced hearing loss: updates on molecular targets and potential interventions. Neural Plast (2021) 2021:4784385. doi: 10.1155/2021/4784385 34306060 PMC8279877

[187] Yang S Weiner BD Zhang LS Cho SJ Bao S. Homeostatic plasticity drives tinnitus perception in an animal model. Proc Natl Acad Sci U.S.A. (2011) 108:14974–9. doi: 10.1073/pnas.1107998108 PMC3169130 21896771

[188] Schaette R Kempter R. Development of tinnitus-related neuronal hyperactivity through homeostatic plasticity after hearing loss: a computational model. Eur J Neurosci (2006) 23:3124–38. doi: 10.1111/j.1460-9568.2006.04774.x 16820003

[189] Salvi RJ Wang J Ding D. Auditory plasticity and hyperactivity following cochlear damage. Hear Res (2000) 147:261–74. doi: 10.1016/S0378-5955(00)00136-2 10962190

[190] Auerbach BD Rodrigues PV Salvi RJ. Central gain control in tinnitus and hyperacusis. Front Neurol (2014) 5:206. doi: 10.3389/fneur.2014.00206 25386157 PMC4208401

[191] Zheng Y McPherson K Smith PF. Effects of early and late treatment with L-baclofen on the development and maintenance of tinnitus caused by acoustic trauma in rats. Neuroscience (2014) 258:410–21. doi: 10.1016/j.neuroscience.2013.11.032 24291770

[192] Cerrah Gunes M Gunes MS Vural A Aybuga F Bayram A Bayram KK. Change in gene expression levels of GABA, glutamate and neurosteroid pathways due to acoustic trauma in the cochlea. J Neurogenet (2021) 35:45–57. doi: 10.1080/01677063.2021.1904922 33825593

[193] Szczepaniak WS Møller AR. Evidence of decreased GABAergic influence on temporal integration in the inferior colliculus following acute noise exposure: a study of evoked potentials in the rat. Neurosci Lett (1995) 196:77–80. doi: 10.1016/0304-3940(95)11851-M 7501262

[194] Middleton JW Kiritani T Pedersen C Turner JG Shepherd GM Tzounopoulos T. Mice with behavioral evidence of tinnitus exhibit dorsal cochlear nucleus hyperactivity because of decreased GABAergic inhibition. Proc Natl Acad Sci U.S.A. (2011) 108:7601–6. doi: 10.1073/pnas.1100223108 PMC3088638 21502491

[195] Browne CJ Morley JW Parsons CH. Tracking the expression of excitatory and inhibitory neurotransmission-related proteins and neuroplasticity markers after noise induced hearing loss. PloS One (2012) 7:e33272. doi: 10.1371/journal.pone.0033272 22428005 PMC3299769

[196] Dong S Mulders WH Rodger J Woo S Robertson D. Acoustic trauma evokes hyperactivity and changes in gene expression in Guinea-pig auditory brainstem. Eur J Neurosci (2010) 31:1616–28. doi: 10.1111/j.1460-9568.2010.07183.x 20525074

[197] Kaltenbach JA Zhang J Finlayson P. Tinnitus as a plastic phenomenon and its possible neural underpinnings in the dorsal cochlear nucleus. Hearing Res (2005) 206:200–26. doi: 10.1016/j.heares.2005.02.013 16081009

[198] Henton A Tzounopoulos T. What's the buzz? The neuroscience and the treatment of tinnitus. Physiol Rev (2021) 101:1609–32. doi: 10.1152/physrev.00029.2020 PMC8576365 33769102

[199] Wu C Stefanescu RA Martel DT Shore SE. Tinnitus: Maladaptive auditory-somatosensory plasticity. Hearing Res (2016) 334:20–9. doi: 10.1016/j.heares.2015.06.005 PMC4676957 26074307

[200] Shore SE Roberts LE Langguth B. Maladaptive plasticity in tinnitus–triggers, mechanisms and treatment. Nat Rev Neurol (2016) 12:150–60. doi: 10.1038/nrneurol.2016.12 PMC4895692 26868680

[201] Haragopal H Dorkoski R Pollard AR Whaley GA Wohl TR Stroud NC. Specific loss of neural sensitivity to interaural time difference of unmodulated noise stimuli following noise-induced hearing loss. J Neurophysiol (2020) 124:1165–82. doi: 10.1152/jn.00349.2020 PMC7717161 32845200

[202] Liberman MC Kujawa SG. Cochlear synaptopathy in acquired sensorineural hearing loss: Manifestations and mechanisms. Hear Res (2017) 349:138–47. doi: 10.1016/j.heares.2017.01.003 PMC5438769 28087419

[203] Scheidt RE Kale S Heinz MG. Noise-induced hearing loss alters the temporal dynamics of auditory-nerve responses. Hear Res (2010) 269:23–33. doi: 10.1016/j.heares.2010.07.009 20696230 PMC2934744

[204] Bakay WMH Anderson LA Garcia-Lazaro JA McAlpine D Schaette R. Hidden hearing loss selectively impairs neural adaptation to loud sound environments. Nat Commun (2018) 9:4298. doi: 10.1038/s41467-018-06777-y 30327471 PMC6191434

[205] Eggermont JJ Roberts LE. The neuroscience of tinnitus. Trends Neurosci (2004) 27:676–82. doi: 10.1016/j.tins.2004.08.010 15474168

[206] Roberts LE Eggermont JJ Caspary DM Shore SE Melcher JR Kaltenbach JA. Ringing ears: the neuroscience of tinnitus. J Neurosci (2010) 30:14972–9. doi: 10.1523/JNEUROSCI.4028-10.2010 PMC3073522 21068300

[207] Eggermont JJ Roberts LE. Tinnitus: animal models and findings in humans. Cell Tissue Res (2015) 361:311–36. doi: 10.1007/s00441-014-1992-8 PMC4487353 25266340

[208] Wang H Brozoski TJ Caspary DM. Inhibitory neurotransmission in animal models of tinnitus: maladaptive plasticity. Hear Res (2011) 279:111–7. doi: 10.1016/j.heares.2011.04.004 PMC3172385 21527325

[209] Zheng Y Vagal S McNamara E Darlington CL Smith PF. A dose-response analysis of the effects of L-baclofen on chronic tinnitus caused by acoustic trauma in rats. Neuropharmacology (2012) 62:940–6. doi: 10.1016/j.neuropharm.2011.09.027 22005094

[210] Yang G Lobarinas E Zhang L Turner J Stolzberg D Salvi R. Salicylate induced tinnitus: behavioral measures and neural activity in auditory cortex of awake rats. Hear Res (2007) 226:244–53. doi: 10.1016/j.heares.2006.06.013 16904853

[211] Butt S Ashraf F Porter LA Zhang H. Sodium salicylate reduces the level of GABAB receptors in the rat's inferior colliculus. Neuroscience (2016) 316:41–52. doi: 10.1016/j.neuroscience.2015.12.021 26705739

[212] Westerberg BD Roberson JB Jr. Stach BA. A double-blind placebo-controlled trial of baclofen in the treatment of tinnitus. Am J Otol (1996) 17:896–903. 8915419

[213] Smith PF Zheng Y Darlington CL. Revisiting baclofen for the treatment of severe chronic tinnitus. Front Neurol (2012) 3:34. doi: 10.3389/fneur.2012.00034 22408636 PMC3297816

[214] Sand PG Langguth B Itzhacki J Bauer A Geis S Cardenas-Conejo ZE. Resequencing of the auxiliary GABA(B) receptor subunit gene KCTD12 in chronic tinnitus. Front Syst Neurosci (2012) 6:41. doi: 10.3389/fnsys.2012.00041 22654739 PMC3360237

[215] Møller AR. Sensorineural tinnitus: its pathology and probable therapies. Int J Otolaryngol (2016) 2016:2830157. doi: 10.1155/2016/2830157 26977153 PMC4761664

[216] Peixoto Pinheiro B Vona B Löwenheim H Rüttiger L Knipper M Adel Y. Age-related hearing loss pertaining to potassium ion channels in the cochlea and auditory pathway. Pflugers Archiv Eur J Physiol (2021) 473:823–40. doi: 10.1007/s00424-020-02496-w PMC8076138 33336302

[217] Caspary DM Ling L Turner JG Hughes LF. Inhibitory neurotransmission, plasticity and aging in the mamMalian central auditory system. J Exp Biol (2008) 211:1781–91. doi: 10.1242/jeb.013581 PMC2409121 18490394

[218] Meredith MA Allman BL Keniston LP Clemo HR. Auditory influences on non-auditory cortices. Hear Res (2009) 258:64–71. doi: 10.1016/j.heares.2009.03.005 19303926 PMC2787633

[219] Shore SE Zhou J. Somatosensory influence on the cochlear nucleus and beyond. Hear Res (2006) 216-217:90–9. doi: 10.1016/j.heares.2006.01.006 16513306

[220] Jovanovic N Suchankova S Kang M Melichar A Bures Z Turecek R. Altered hearing function in mice with implanted cranial windows. Neurosci Lett (2023) 792:136969. doi: 10.1016/j.neulet.2022.136969 36402256

[221] Paciello F Rinaudo M Longo V Cocco S Conforto G Pisani A. Auditory sensory deprivation induced by noise exposure exacerbates cognitive decline in a mouse model of Alzheimer's disease. Elife (2021) 10:e70908. doi: 10.7554/eLife.70908 34699347 PMC8547960

[222] Abidin FNZ Wells HRR Altmann A Dawson SJ. Hearing difficulty is linked to Alzheimer's disease by common genetic vulnerability, not shared genetic architecture. NPJ Aging Mech Dis (2021) 7:17. doi: 10.1038/s41514-021-00069-4 34294723 PMC8298411

[223] Nadhimi Y Llano DA. Does hearing loss lead to dementia? A Rev literature. Hear Res (2021) 402:108038. doi: 10.1016/j.heares.2020.108038 PMC9336511 32814645

[224] Goble TJ Moller AR Thompson LT. Acute high-intensity sound exposure alters responses of place cells in hippocampus. Hear Res (2009) 253:52–9. doi: 10.1016/j.heares.2009.03.002 19303432

[225] Griffiths TD Lad M Kumar S Holmes E McMurray B Maguire EA. How can hearing loss cause dementia? Neuron (2020) 108:401–12. doi: 10.1016/j.neuron.2020.08.003 PMC7664986 32871106

[226] Tarawneh HY Jayakody DMP Sohrabi HR Martins RN Mulders W. Understanding the relationship between age-related hearing loss and alzheimer's disease: A narrative review. J Alzheimers Dis Rep (2022) 6:539–56. doi: 10.3233/ADR-220035 PMC9535607 36275417

[227] Ferland JN Carr MR Lee AM Hoogeland ME Winstanley CA Pattij T. Examination of the effects of cannabinoid ligands on decision making in a rat gambling task. Pharmacol Biochem Behav (2018) 170:87–97. doi: 10.1016/j.pbb.2018.05.012 29787777

[228] Helm KA Haberman RP Dean SL Hoyt EC Melcher T Lund PK. GABAB receptor antagonist SGS742 improves spatial memory and reduces protein binding to the cAMP response element (CRE) in the hippocampus. Neuropharmacology (2005) 48:956–64. doi: 10.1016/j.neuropharm.2005.01.019 15857622

[229] Porcu A Melis M Turecek R Ullrich C Mocci I Bettler B. Rimonabant, a potent CB1 cannabinoid receptor antagonist, is a Galpha(i/o) protein inhibitor. Neuropharmacology (2018) 133:107–20. doi: 10.1016/j.neuropharm.2018.01.024 29407764

[230] Sereikaite V Fritzius T Kasaragod VB Bader N Maric HM Schindelin H. Targeting the gamma-aminobutyric acid type B (GABA(B)) receptor complex: development of inhibitors targeting the K(+) channel tetramerization domain (KCTD) containing proteins/GABA(B) receptor protein-protein interaction. J Med Chem (2019) 62:8819–30. doi: 10.1021/acs.jmedchem.9b01087 31509708

[231] Shaye H Stauch B Gati C Cherezov V. Molecular mechanisms of metabotropic GABA(B) receptor function. Sci Adv (2021) 7:eabg3362. doi: 10.1126/sciadv.abg3362 34049877 PMC8163086

